# Gene synteny comparisons between different vertebrates provide new insights into breakage and fusion events during mammalian karyotype evolution

**DOI:** 10.1186/1471-2148-9-84

**Published:** 2009-04-24

**Authors:** Claus Kemkemer, Matthias Kohn, David N Cooper, Lutz Froenicke, Josef Högel, Horst Hameister, Hildegard Kehrer-Sawatzki

**Affiliations:** 1 Institute of Human Genetics, University of Ulm, 89081 Ulm, Germany; 2 Institute of Medical Genetics, School of Medicine, Cardiff University, Cardiff, UK; 3 Dept. of Population Health and Reproduction, School of Veterinary Medicine, University of California, Davis, CA 95616, USA; 4 LMU München, Biozentrum Martinsried, München, Germany

## Abstract

**Background:**

Genome comparisons have made possible the reconstruction of the eutherian ancestral karyotype but also have the potential to provide new insights into the evolutionary inter-relationship of the different eutherian orders within the mammalian phylogenetic tree. Such comparisons can additionally reveal (i) the nature of the DNA sequences present within the evolutionary breakpoint regions and (ii) whether or not the evolutionary breakpoints occur randomly across the genome. Gene synteny analysis (E-painting) not only greatly reduces the complexity of comparative genome sequence analysis but also extends its evolutionary reach.

**Results:**

E-painting was used to compare the genome sequences of six different mammalian species and chicken. A total of 526 evolutionary breakpoint intervals were identified and these were mapped to a median resolution of 120 kb, the highest level of resolution so far obtained. A marked correlation was noted between evolutionary breakpoint frequency and gene density. This correlation was significant not only at the chromosomal level but also sub-chromosomally when comparing genome intervals of lengths as short as 40 kb. Contrary to previous findings, a comparison of evolutionary breakpoint locations with the chromosomal positions of well mapped common fragile sites and cancer-associated breakpoints failed to reveal any evidence for significant co-location. Primate-specific chromosomal rearrangements were however found to occur preferentially in regions containing segmental duplications and copy number variants.

**Conclusion:**

Specific chromosomal regions appear to be prone to recurring rearrangement in different mammalian lineages ('breakpoint reuse') even if the breakpoints themselves are likely to be non-identical. The putative ancestral eutherian genome, reconstructed on the basis of the synteny analysis of 7 vertebrate genome sequences, not only confirmed the results of previous molecular cytogenetic studies but also increased the definition of the inferred structure of ancestral eutherian chromosomes. For the first time in such an analysis, the opossum was included as an outgroup species. This served to confirm our previous model of the ancestral eutherian genome since all ancestral syntenic segment associations were also noted in this marsupial.

## Background

By comparison with other vertebrates, mammals display a high degree of karyotype variability. Chromosome numbers vary considerably, ranging from 2n = 6 in the Indian muntjak [1] to 2n = 102 in the red viscacha rat [2]. Despite this numerical variability, conserved (syntenic) chromosome segments have been successfully identified by means of comparative cytogenetics [3]. A conserved genome framework, initially concealed by inter-species karyotypic divergence, was first revealed by comparative gene mapping, but became readily apparent with the advent of comparative chromosome painting. The application of these methodologies has served to confirm the presence of a limited number of chromosomal segments which have been evolutionarily conserved across a variety of mammalian species [4-7]. Taken together, these approaches have allowed the reconstruction of synteny maps of a number of ancestral mammalian genomes [8-12].

Recently performed comparisons of entire genome sequences have extended our understanding of the evolutionary history of mammalian genomes by revealing the presence of a limited number of syntenic segments with highly conserved gene orders, termed 'conserved linkage groups' [9,13-16]. These segments can be used, almost as if they were pieces of a giant jig-saw puzzle, to compare extant genomes as well as to reconstruct ancestral genomes. Both comparative chromosome painting and genome sequence comparisons have indicated that the human genome possesses an organization which is highly conserved evolutionarily and which displays considerable similarity to the postulated ancestral eutherian karyotype [10,12,17] dating from ~105 million years ago (MYA) [18].

Ancestral genome models deduced from comparative cytogenetic analysis exhibit marked differences when compared to reconstructions of ancestral eutherian genomes based on whole genome sequence alignments [19,20]. Recently, we devised a simplified method of comparative genome analysis based on the comparison of gene order in different species. By focussing exclusively on the relative positions of genes instead of aligning large contigs of genomic DNA, this method reduces the complexity of whole genome alignments thereby facilitating the identification of conserved syntenic segments. This technique was used successfully to identify the evolutionary origin of the mammalian X chromosome from three distinct ancestral chromosome building segments [21] and has also made possible the reconstruction of a vertebrate protokaryotype from 450 MYA [22]. Since this methodology relies upon *in silico *gene order comparisons using genome sequence data from different species, an approach reminiscent of comparative chromosome painting, the *in silico *approach has been termed 'E-painting' (electronic chromosome painting) [22].

Estimates of the number, location and extent of evolutionary breakpoint intervals vary owing to methodological differences, and this variation has helped to fuel considerable controversy. Recent comparative genome sequence studies have been interpreted as indicating that evolutionary chromosomal rearrangements are non-randomly distributed across mammalian genomes and that the associated breakpoints have often been 'reused' [9,23,24]. The resulting 'fragile breakage model' of genome evolution has therefore presented a direct challenge to the now classical 'random breakage' model of Nadeau and Taylor [25].

In this study, we have performed an *in silico *genome-wide analysis of synteny (E-painting) in order to improve our understanding of the organization of the ancestral eutherian genome. Our analysis employed genome sequence data from human [26], mouse [27], rat [28], dog [29], cow ; B_tau3.1, and opossum [30], genomes which have all been sequenced with at least 7-fold coverage. The chicken genome sequence [31] was also included in our comparison since previous studies have shown that chicken genome organization displays a remarkable resemblance to that of eutherian mammals [9,21] despite its evolutionary divergence about 310 MYA.

## Results

### Establishment of syntenic relationships and reconstruction of ancestral karyotypes

A previous comparative synteny analysis of about 3000 human genes and their orthologues in 5 other vertebrate species permitted the first reconstruction of an ancestral vertebrate karyotype [22]. In this study, we have extended this comparative approach to identifying syntenic segments of orthologous genes and included all those human genes for which orthologues have been annotated in the genomes of mouse, rat, dog, cow, opossum (a marsupial) and chicken. Beginning with 28197 human genes (Human Genome Assembly 18, NCBI build 36), the number of orthologous genes in the studied species identified by the program BioMart ranges from 12591 in chicken to 17796 in mouse (Table 1). The maximum number of orthologous genes identifiable in a given species (by comparison with human) was recruited on the basis that the higher the number of genes employed in the analysis, the more precise would be the identification of the breakpoint intervals. Had we considered only those genes for which a one-to-one orthology relationship was identifiable in all species under investigation, this would have resulted in a considerable decrease in the number of genes to be analysed and hence a substantial decrease in the degree of resolution possible. Instead, the genome-wide coverage attained by using the maximum number of orthologous genes identifiable between human and the other studied vertebrate species served to optimize the resolution of the mapping of the evolutionary chromosomal breakpoints.

**Table 1:** Number of genes in different species for which unambiguous orthologies to a total of 28197 annotated human genes were identified using the BioMart program.

**Species (abbreviation)**	**Number of genes orthologous to 28197 annotated human genes^a^**
*Rattus norvegicus (RNO)*	17733

*Mus musculus (MMU)*	17796

*Canis familiaris (CFA)*	16263

*Bos taurus (BTA)*	16689

*Monodelphis domestica (MDO)*	15257

*Gallus gallus (GGA)*	12591

a: Orthologous genes were determined using the program BioMart

(; ENSEMBL release 46).

**Figure 1:**
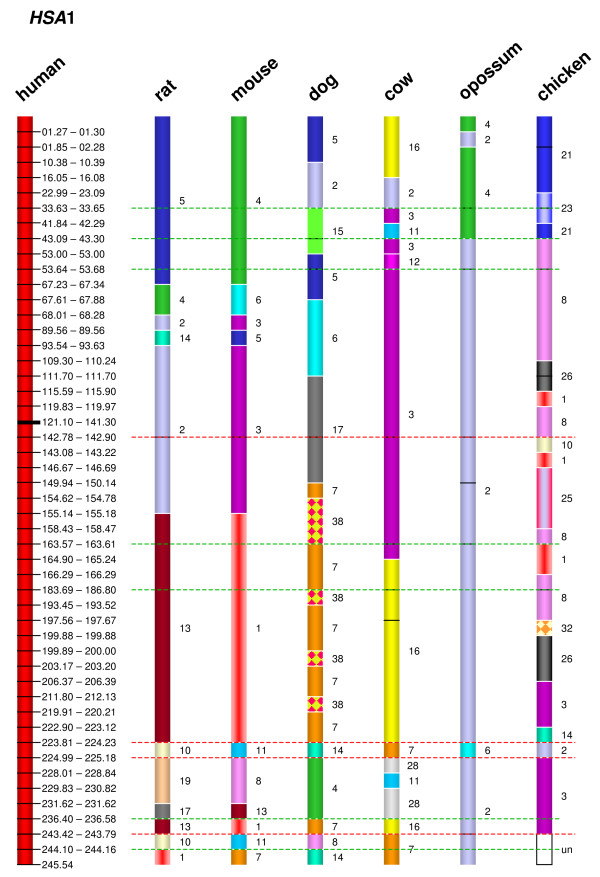
**Ideogram of human chromosome 1 (*HSA*1) and its orthologues as determined by E-painting in rat, mouse, dog, cow, opossum and chicken**. The human chromosome coordinates of the breakpoint intervals are given to the right of the human ideogram in Mb. The chromosome number of the orthologous segments in the analyzed species is indicated to the right of each conserved segment. Chromosomal breakpoints have been evenly spaced in order to optimize visualization of the conserved syntenic segments. The resulting ideograms of the chromosomes and conserved segments are therefore not drawn to scale. The centromeric region is indicated by a black horizontal bar on the human ideogram. The stippled red lines indicate breaks present in all analyzed non-human genomes and which may thus be attributable to rearrangements specific to the primate lineage (see Table 3). Black lines within the ideograms indicate breaks within the contiguous sequence that probably resulted from intrachromosomal rearrangements caused by inversions. Stippled green lines indicate the positions of 'reused breakpoints', defined as locations in which breakpoints were found to map to the same genomic intervals in at least three species from two different clades. The complete set of E-painting results for chromosomes 1–22 is given in Additional file 1. un: undetermined.

**Figure 2:**
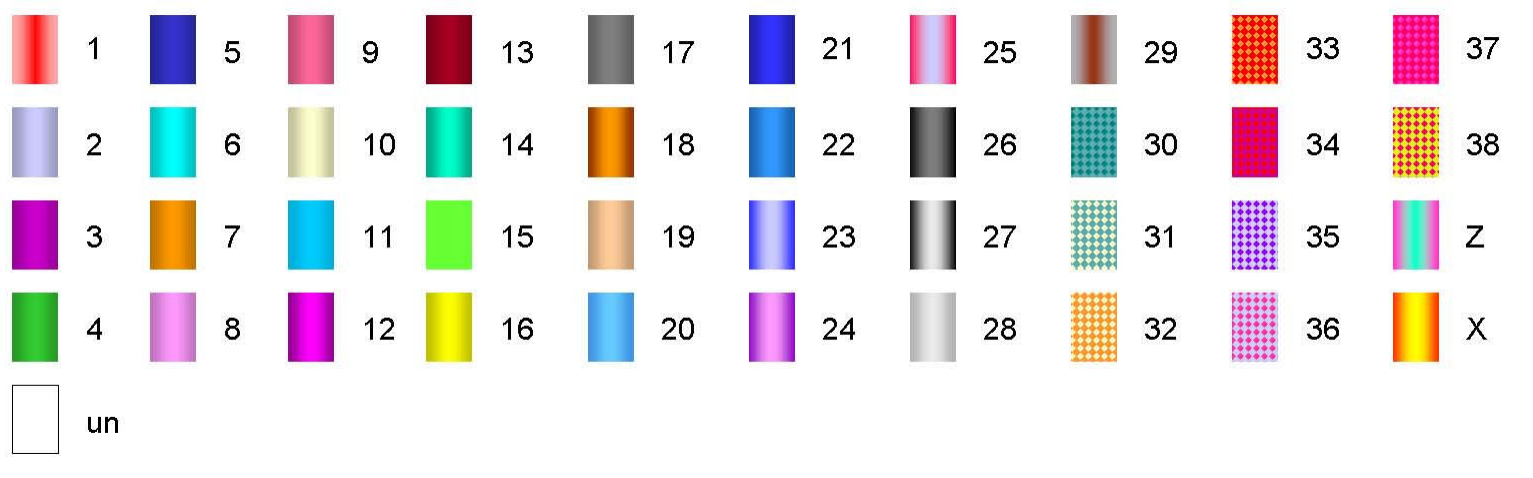
**The colour code for chromosomal regions 1–38, X and Z chromosomes was employed to indicate regions of conserved synteny in Figure 1 and Additional file **1. The same colour code was also used to depict the ancestral boreoeutherian karyotype indicated in Figure 3.

**Figure 3:**
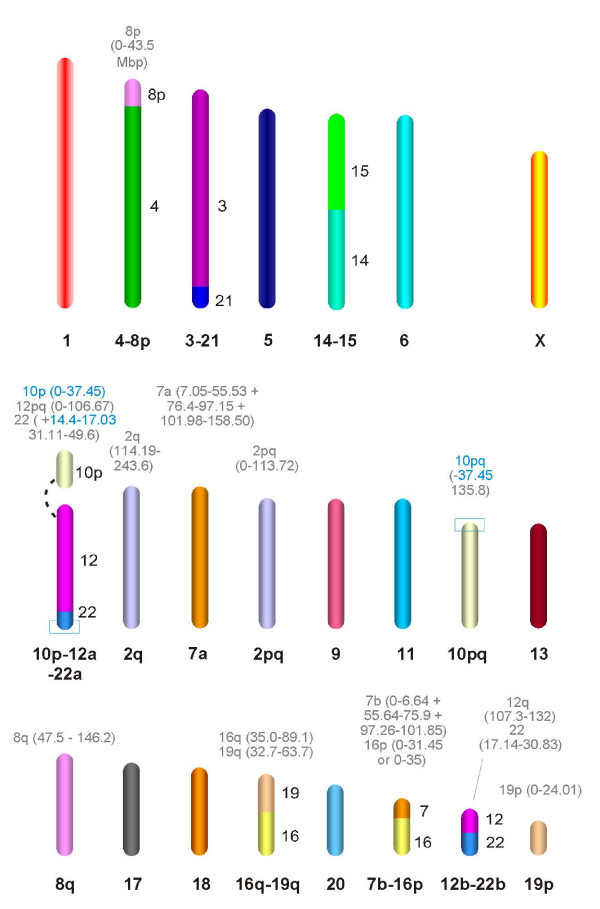
**The reconstructed ancestral boreoeutherian karyotype, derived from synteny analyses of human, mouse, rat, cow, dog, opossum and chicken genome sequences, and based on the identified orthology blocks, is depicted in Additional file **1. The ideograms represent the 22 autosomal syntenic groups of the ancestral genome as well as the ancestral X chromosome. The orthologies to the human genome are given for entire chromosomes below each chromosomal ideogram and to the right of the ideograms for the individual conserved segments. For conserved segments representing portions of human chromosomes, the positions of the boundaries of the orthologous segments in the human genome are listed above the ideograms in Mb. Boundaries in agreement with previous findings, and based on comparative cytogenetics, are given in black whereas the boundaries refined in this study are indicated in blue. The sizes of the chromosomal ideograms reflect the approximate size ratios of the euchromatic orthologous segments in the human genome. The association of the segment orthologous to *HSA*10p with segments orthologous to *HSA*12 and *HSA*22 is based on comparative chromosome painting data from carnivores [61], hedgehog, several afrotherian [10,60] and xenarthran [55,56] species as well as the opossum genome sequence [30]. The comparative chromosome painting data for afrotherian and xenarthran species further indicate that the syntenic groups of the ancestral boreoeutherian karyotype are identical with those of the eutherian karyotype.

The dataset from human, representing the best characterized vertebrate genome to date (as well as one of the evolutionarily most conserved karyotypes among eutherian mammals), provided the reference against which segments of conserved syntenic genes could be identified in the genomes of the other species under investigation. In principle, blocks or segments containing syntenic human genes were sought which are also present as blocks of syntenic genes in the other species under study. Conversion of the syntenic segment associations into colour-coded ideograms rendered the conserved syntenic segments (and at the same time, the breakpoint intervals) readily identifiable (Figure 1; Additional file 1). The colour code employed in Figure 2 was used to indicate the orthologous relationships of syntenic segments in a comparison of the different species with human, as depicted in Figure 1, Additional file 1 and Figure 3. For example, the region of human chromosome 1 between positions 1.27 Mb and 67.23 Mb is identifiable as a continuous (syntenic) segment on rat chromosome 5 and mouse chromosome 4 (Figure 1). During our analysis, we considered as evolutionary breakpoints those disruptions in gene order (synteny) that resulted from (i) interchromosomal rearrangements in an ancestral species as deduced by comparing human with one of the other six species under investigation and (ii) intrachromosomal inversions that occurred in the human lineage where both breakpoint regions could be identified. If the breakpoint region of an interchromosomal rearrangement, identified by comparing the human genome with that of another species, was found to coincide with the breakpoint of an intrachromosomal rearrangement in any one of the other species, this intrachromosomal breakpoint was also considered as a break in synteny.

Employing these criteria to define evolutionary breakpoint intervals, a total of 526 such intervals, with an average size of 290 kb and a median size of 120 kb, were identified (Table 2; Additional file 2). To visualize all syntenic breakpoint intervals, chromosome ideograms were drawn up such that all breakpoints were arranged equidistantly, with the precise positions of the breakpoint intervals being demarcated by the genomic coordinates of the flanking genes (an exemplar is shown in Figure 1 for *HSA*1, whilst all ideograms from chromosomes 1 to 22 are depicted in Additional file 1). The orthologous relationships between the analyzed genomes served to identify a total of 38 different ancestral syntenic segments which are indicated by a colour code in Figure 2. The ideograms in Figure 1 and Additional file 1 are equivalent to a reverse chromosome painting dataset of the six analyzed species onto human chromosomes at high resolution. The precise positions of the genes flanking all identified breakpoint intervals are listed in Additional file 2.

**Table 2:** Number of evolutionary breakpoint intervals per chromosome and their characteristics.

**Chromosome**	**Number of ** **intervals per** **breakpoint** **chromosome**	**Length of all ** **breakpoint ** **intervals in Mb ** **(% whole chromosome)**	**Median length ** **of breakpoint** **interval (Mb)**	**Average length ** **of breakpoint** **interval (Mb)**	**Gene density, ** **entire chromosome** **(genes/Mb)**
**1**	47	11.89 (4.84)	0.11	0.25	8.67

**2**	46	17.87 (7.34)	0.15	0.40	5.66

**3**	45	13.60 (6.82)	0.14	0.30	5.58

**4**	21	8.78 (4.57)	0.12	0.42	4.35

**5**	42	18.24 (10.09)	0.17	0.43	5.11

**6**	28	6.24 (3.67)	0.12	0.23	6.48

**7**	37	8.21 (5.18)	0.11	0.23	6.25

**8**	30	9.94 (6.80)	0.21	0.34	5.06

**9**	20	6.31 (4.56)	0.23	0.33	6.54

**10**	20	7.39 (5.44)	0.11	0.39	6.05

**11**	31	8.58 (6.37)	0.16	0.28	10.36

**12**	17	3.06 (2.32)	0.13	0.18	8.34

**13**	9	2.32 (2.03)	0.14	0.26	3.14

**14**	11	2.87 (2.69)	0.11	0.26	6.23

**15**	16	2.12 (2.10)	0.06	0.13	6.57

**16**	12	4.21 (4.73)	0.03	0.38	10.33

**17**	31	5.63 (7.13)	0.09	0.19	15.70

**18**	11	7.71 (10.16)	0.59	0.77	3.86

**19**	17	1.49 (2.34)	0.05	0.09	22.47

**20**	20	3.21 (5.14)	0.07	0.17	9.72

**21**	6	0.46 (0.98)	0.04	0.08	5.79

**22**	9	1.60 (3.23)	0.12	0.18	10.28

**sum**	**526**	**151.73**	**0.12**	**0.29**	

The graphical compilation of syntenic disruptions shown in Additional file 1 indicates that 7.6% of the evolutionary breakpoints (N = 40 of 526, highlighted by stippled green lines) have been 'reused' i.e. breakpoints were found in the same genomic intervals in at least three species from two different clades (reused breakpoints are marked in red in Additional file 1). The assignment of the species under investigation to different clades within the mammalian phylogenetic tree is indicated in Additional file 3 (during this analysis, chicken and opossum were considered as two different clades). Taking all autosomes into consideration, 218 breakpoint regions were identified in a comparison of the chicken and human genomes whereas 153 breaks in synteny serve to differentiate the human and opossum chromosomes. A total of 27 breakpoints were found to be shared between chicken and opossum but were not observed in any other species, suggesting that these constitute evolutionary breakpoints that occurred in the eutherian common ancestor (Additional file 2). A comparison of the gene orders exhibited by both murid species with those of humans, revealed 106 breaks in synteny (Additional file 2). However, only 4 breaks in synteny were specific to the rat whereas 17 were specific to the mouse. The many murid-shared breaks in synteny (N = 85) as compared with humans is clearly a reflection of the extended common phylogenetic history of mouse and rat, which only became separated into distinct species 16–23 MYA [32,33]. The two ferungulate species, dog and cow, only share 14 breaks, with 65 breaks being restricted to the canine lineage and 114 breaks confined to the bovine lineage [34]. The much higher number of lineage-specific breaks in these two species, both of which belong to the Laurasiatheria, is indicative of the longer period of time that has elapsed since the evolutionary divergence of the carnivores and artiodactyls ~88 MYA [18].

The version of the cow genome used for our analysis (Btau_3.1) may contain some local errors caused by intrachromosomal misplacement of scaffold. These intrachromosomal inconsistencies are not however relevant to the tests we have performed since we were primarily interested in analysing interchromosomal rearrangements between the human and bovine genomes.

Several breaks in synteny were identified in mouse, rat, dog, cow, opossum and chicken that are common to all six species (Additional file 2). The most parsimonious explanation for this observation is not breakpoint 'reuse' but rather that these were primate- (or even human-) specific breaks. Some 63 such primate lineage-specific breakpoints were identified and these are indicated by stippled red lines in the ideograms (Fig. 1A, Additional file 1). Most of these breaks appear to have been caused by primate-specific inversions (N = 22, Table 3). Proportional to its length, *HSA*17 is especially rich in such primate-specific inversions. A disproportionate number of these inversions were also noted in the orthologous segment of *HSA*19p in the lineage leading to rodents, in the orthologous segment of *HSA*20p in the lineage leading to chicken and in the orthologous segment of *HSA*1 in the canine lineage (Additional file 1). The remaining primate-specific breakpoints may be attributable to chromosome fusions and insertions of small segments.

**Table 3:** Summary of the evolutionary breakpoint intervals specific to the primate lineage.

**Breakpoint interval number**	**Chromosome**	**5'boundary of the breakpoint interval (Mb)**	**3'boundary of the breakpoint interval (Mb)**	**Inversion^a^**
1	1	142.78	142.90	

2	1	223.81	224.23	4^1^

3	1	224.99	225.18	

4	1	243.42	243.79	2^1^

5	2	50.49	53.81	

6	2	cent	cent	7^2^

7	2	110.02	110.20	6^2^

8	2	113.72	114.19	9^3^

9	2	132.07	133.00	8^3^

10^a^	3	ptel	ptel	11^4^

11	3	12.86	13.00	10^4,5,6^,14^4,5,6^,16^4,5,6^

12	3	15.12	15.22	

13	3	74.62	77.17	

14	3	127.18	127.28	11^5,7^,17^5,7^

15	3	130.52	130.58	

16	3	131.18	131.64	11^6^

17^a^	3	qtel	199.33	14^7^

18	5	147.57	147.8	

19	7	6.64	7.05	22^8^

20	7	55.53	55.64	

21	7	75.90	76.40	23^9^

22	7	97.15	97.26	19^8^

23	7	101.85	101.98	21^9^

24	8	7.87	8.21	26^10,11^,29^10,11^

25	8	9.68	9.95	28^12^

26	8	11.76	12.03	24^10^

27	8	17.99	18.11	

28	8	29.18	29.25	25^12^

29	8	cent	cent	24^11^

30	9	38.41	39.06	

31	9	91.79	92.05	

32	9	94.17	94.4	

33	9	96.88	97.08	

34	10	27.57	27.83	

35	10	35.97	37.45	

36	10	51.56	51.62	37^13^

37	10	88.94	89.25	36^13^

38	11	3.20	3.62	39^14^

39	11	70.94	71.32	38^14^

40	12	106.67	107.03	

41	13	40.14	40.40	42^15^

42	13	52.06	52.12	41^15^

43	15	26.24	27.00	

44	15	30.57	30.69	46^16^

45	15	76.02	76.07	

46	15	100.08	100.16	44^16^

47	16	31.45	31.98	

48	16	cent	cent	

49^a^	17	ptel	ptel	54^17^

50	17	6.68	6.84	51^18^

51	17	15.58	15.74	50^18^

52	17	16.65	16.78	53^19^

53	17	20.31	20.84	52^19^

54	17	cent	cent	49^17,20^,56^17,20^

55	17	25.88	26.08	

56	17	26.31	26.45	54^20^

57	17	33.18	33.71	58^21^

58	17	57.50	57.81	57^21^

59	18	39.11	40.54	

60	19	6.94	6.97	61^22^

61	19	8.70	8.79	60^22^

62	19	15.97	16.04	

63	19	cent	cent	

64	22	17.03	17.14	

65	22	23.31	23.44	

66	22	30.83	31.11	

^a^: These 3 inversion breakpoint regions were deduced, from the human genome sequence, to harbour the second breakpoint of the respective primate-specific inversions. They were, however, not evident as such in the sequences of the 6 species under investigation (mouse, rat, dog, cow, chicken and opossum) and hence are not indicated in Additional files 1 and 2.

^b^: The superscript numbers in the last column indicate putative inversions. Thus, chromosomes orthologous to *HSA *4, 6, 14, 20 and 21 were not rearranged in the primate lineage.

Employing the previously described method of concatenating overlapping conserved syntenic segments [34], the eutherian mammal genome data permitted the seamless assembly of conserved segments into ancestral chromosomes. Ancestral associations between conserved syntenic segments are identifiable by virtue of the presence of shared orthologies between mammalian chromosomes from at least three different species. The resulting model of the ancestral boreoeutherian genome (Figure 3), with a chromosome number of 2n = 46, describes the karyotype of the last common ancestor of primates and rodents (superorder Euarchontoglires, Additional file 3) as well as of carnivores and cetartiodactyls (superorder Laurasiatheria).

### Chromosomal sites of syntenic breakage

High precision syntenic breakpoint mapping permits the evaluation, at least in principle, of whether or not these evolutionary breaks coincide with potential hotspots of chromosomal rearrangement such as fragile sites or cancer-associated breakpoints. Fragile sites are classified as either rare (spontaneously occurring) or common (inducible) [35]. Altogether, some 89 common fragile sites have been mapped at the cytogenetic level [36] although only the 11 most common autosomal fragile sites have been precisely characterized at the molecular level [35,37-49]. A comparison of these 11 precisely characterized fragile sites with the positions of the evolutionary breakpoints identified in this study indicated that only FRA4F and FRA7E, which span distances of 5.9 Mb and 4.4 Mb respectively, partially overlap with evolutionary breakpoint regions (Table 4). For none of the other 524 evolutionary breakpoints was any overlap with a fragile site observed. Under a random model, we estimate that ~1.23% (37.9/3093) of the 526 observed breakpoints intervals would have been expected to overlap with one of the 11 fragile sites. Since only 2/526 breakpoints (0.38%) were found to display an overlap with a fragile site (p = 0.11), we concluded that there was no evidence for extensive co-location.

**Table 4:** Autosomal common fragile sites, whose locations on the human genome sequence have been demarcated by flanking markers, and their overlap with evolutionary breakpoint intervals.

**Fragile site [reference]**	**Location in the human genome**				**Overlap with evo breakpoint**
	**5' Boundary**		**3' Boundary**		
		
	**Marker designation**	**Position (Mb)**	**Marker designation**	**Position (Mb)**	

					

FRA2G [38]	*LASSG*	169	*PPJG*	170.2	--

FRA3B [39]	SHGC86352	59.7	RH41625	60.5	--

FRA4F [40]	*SNCA*	90.8	*MNC5C*	96.7	95.62–95.73

FRA6E [41]	D6S1581	160.2	D6S1719	165.9	--

FRA6F [42]	SHGC144121	111.7	SHGC82095	112.7	--

FRA7E [43]	D7S1934	80.3	SHGC104456	84.7	84.40–85.92

FRA7G [44]	SHGC143971	115.6	RH44861	116.2	--

FRA7H [45]	D7S614	130.2	STSG33535FS	130.4	--

FRA7I [46]	SHGC153624	144.3	SWSS2627	145.7	--

FRA7K [47]	*IMMP22L*	109.9	*IMMP22L*	110.7	--

FRA9E [48]	D9S1866	106.9	D9S154	118.4	--

FRA16D [49]	SHGC150973	76.7	WJ2755	77.8	--

A second class of chromosomal breakage hotspot is represented by recurring cancer-associated breakpoints. Although the majority of such breakpoints have been assigned to cytogenetic bands, they have not yet been mapped with any degree of precision. A variety of genes, with actual or potential roles in tumorigenesis, nevertheless reside at or near these breakpoints. We therefore identified the exact genomic positions of 387 annotated cancer-associated autosomal genes using the *Atlas of Genetics and Cytogenetics in Oncology and Haematology *. For the purposes of this analysis, only well-established cancer-associated genes were included (for convenience, these are listed separately in this database). Other genes in this database that have not yet been convincingly implicated in cancer were not included in this analysis. Of the 387 cancer genes, only 13 mapped to evolutionary breakpoint intervals identified in this study (Table 5, Additional file 2). Since the 526 evolutionary breakpoint intervals together comprise 151.7 Mb of genomic sequence, we estimate that some 20 cancer-associated genes might have been expected to occur within the breakpoint intervals by chance alone. We therefore conclude that genes occurring at cancer-associated breakpoints are not disproportionately represented within regions of evolutionary breakpoints.

**Table 5:** Evolutionary breakpoint intervals 'co-localizing' with known cancer-associated genes.

**Human chromosome**	**Evolutionary breakpoint interval ^a^**		**Cancer-associated genes located within the respective evolutionary breakpoint interval ^b^**	**Species exhibiting the corresponding evolutionary break**
	**Position of the 5' boundary (Mb)**	**Position of the 3' boundary (Mb)**		

**1**	1.85	2.28	*PRKCZ, SKI*	*MDO, GGA*

**2**	230.76	231.4	*SP100*	*BTA, MDO, GGA*

**3**	184.47	184.69	*MCF2L2*	*BTA, MDO*

**5**	112.29	112.88	*ZRSR1, MCC*	*MMU, CFA, MDO*

**6**	74.03	74.19	*DDX43*	*RNO, MMU*

**11**	75.60	76.05	*C11orf30*	*BTA*

**11**	93.55	93.92	*MRE11A*	*BTA*

**11**	101.61	101.77	*BIRC3*	*BTA*

**11**	110.09	110.84	*POU2AF1*	*GGA*

**12**	56.26	56.62	*CDK4*	*GGA*

**19**	58.70	59.41	*TFPT*	*MMU*

a: Evolutionary breakpoint intervals are indicated with respect to their position on the orthologous human chromosome.

b: Data on cancer-associated genes were extracted from the *Atlas of Genetics and Cytogenetics in Oncology and Haematology *.

The question then arises as to the location of these evolutionary breakpoints in relation to genes and other DNA sequence features. As mentioned above, a total of 66 primate-specific breaks in synteny were identified in this analysis. Remarkably, 78% of these breakpoint intervals coincide with segmental duplications (SDs) in the human genome (Additional file 2) despite the fact that SDs comprise only 4–5% of the human genome sequence [50-52]. Colocalization with copy number variants (CNVs) was also observed in the case of 76% of these breakpoints (Additional file 2). Thus, primate-specific breakpoint regions would appear to be highly enriched for both SDs and CNVs.

Those human chromosomes that are known to be gene-dense also appear to contain significantly more breakpoints than gene-poor chromosomes (Table 6). Indeed, a strong correlation was noted between protein-coding gene density and the number of evolutionary breakpoints per chromosome (r = 0.60; p = 0.0031). When the gene-dense chromosomes *HSA*17, *HSA*19 and *HSA*22 were directly compared with the gene-poor chromosomes *HSA*13, *HSA*18 and *HSA*21, the gene-dense chromosomes exhibited nearly three times as many breaks per Mb as gene-poor chromosomes.

**Table 6:** Numbers of evolutionary breakpoint intervals on chromosomes 1 – 22, length of the respective chromosomes and gene density.

**Chromosome**	**Number of evolutionary breakpoint intervals per chromosome**	**Chromosome** **length (bp) ^a^**	**Number of protein-coding genes per chromosome ^a^**	**Gene density (genes/100 kb)**	**Breakpoint density (breakpoint interval/100 kb)**
					

1	47	247,249,719	2189	8.85	0.19

2	46	242,951,149	1328	5.47	0.19

3	45	199,501,827	1112	5.57	0.23

4	21	191,273,063	797	4.17	0.11

5	42	180,857,866	903	4.99	0.23

6	28	170,899,992	1133	6.62	0.16

7	37	158,821,424	1023	6.44	0.23

8	30	146,274,826	747	5.11	0.21

9	20	140,273,252	929	6.62	0.14

10	20	135,374,737	834	6.16	0.15

11	31	134,452,384	1385	10.30	0.23

12	18	132,349,534	1080	8.16	0.14

13	9	114,142,980	361	3.16	0.08

14	11	106,368,585	669	6.29	0.10

15	16	100,338,915	641	6.39	0.16

16	12	88,827,254	925	10.41	0.13

17	31	78,774,742	1236	15.69	0.39

18	11	76,117,153	295	3.88	0.14

19	17	63,811,651	1443	22.61	0.27

20	20	62,435,964	617	9.88	0.32

21	6	46,944,323	284	6.05	0.13

22	9	49,691,432	519	10.44	0.18

a: according to the Ensembl database, version 47.36i. The gene density per chromosome was found to be correlated with the number of breakpoint intervals (r = 0.60; p = 0.0031).

We further observed a correlation between transcript density and breakpoint occurrence (r = 0.62, p = 0.0029). To calculate this correlation coefficient, we used the Human Transcriptome Map, based on the draft human genome sequence as provided by the UCSC Genome Bioinformatics Project , which includes all transcribed sequences except processed pseudogenes (according to Versteeg et al. [53]). The correlation noted between transcript density and breakpoint occurrence became even stronger when chromosomal regions were considered rather than entire chromosomes. The evolutionary breakpoint regions identified here exhibited a 1.54-fold increase in transcript density for the central 1 Mb of syntenic breakpoint regions as compared to the genome average (Additional file 4). When this analysis was further restricted to the 144 most precisely mapped breakpoint intervals of <40 kb, the transcript density attained a value some 2.9 times that of the genome-wide average (Additional file 5). Finally, analyses of breakpoint intervals assigned to individual evolutionary lineages indicated that the breakpoint regions identified in both chicken and opossum lineages displayed very high transcript densities corresponding to 3.7 times the genomic average (Table 7).

**Table 7:** Average transcript density of lineage-specific breakpoints observed for regions of 125 kb around the arithmetic centre of the evolutionary breakpoint interval.

**Feature investigated**	**Species**				
	**Chicken**	**Opossum**	**Cow/Dog**	**Mouse/Rat**	**Human**

**No. of evolutionary breakpoint intervals**	83	25	166	120	56

**Average size of the breakpoint interval (Mb)**	0.16	0.24	0.27	0.35	0.39

**Transcripts/Mb**	29.76	27.76	17.52	16.20	20.32

**Increase above the genome average transcript density**	3.72-fold	3.72-fold	2.19-fold	2.03-fold	2.54-fold

NB. Estimates of both the average size of the breakpoint interval and the transcript density are sensitive to the relative-fold sequence coverage of the respective genomes. However, calculating values for the increase above the genome average transcript density allows valid inter-genome comparisons to be made.

### Random breakage or non-random location of evolutionary breakpoints

In order to ascertain whether the evolutionary breakpoints identified in this study occurred randomly or were instead preferentially located in certain genomic regions, we performed simulation experiments. To avoid consideration of breakpoints that did not result from independent breakage (and which could have been identical-by-descent), we selected only breakpoints that were present in mouse, cow, opossum and chicken, respectively. Breakpoints in rat and dog were excluded from this analysis in order to avoid consideration of breakpoints that could have been identical-by-descent and shared either by mouse and rat or by dog and cow. For example, breakpoints present in mouse and rat (as compared to human) could have been identical-by-descent yet would have been counted twice in our analysis. Thus, only breakpoints in mouse and cow were considered (and not those in rat and dog) in order to avoid the potential double-counting of some evolutionary breakpoints. Those 63 breakpoint regions observed in all 4 species (mouse, cow, opossum, chicken) compared to human, and which were thus specific to the primate lineage, were also excluded (indicated in yellow in Additional file 2). Finally, a total of 519 breakpoints were considered that were evident in four species (N = 132 in mouse, N = 143 in cow, N = 89 in opossum and N = 155 in chicken; Additional file 2). These 519 breakpoints occurred in 410 genomic regions, 324 of which contained a breakpoint observed in only one species (as compared to human), whereas 63 genomic regions contained breakpoints in two species, and 23 genomic regions contained breakpoints in three species.

By means of a simulation with 100,000 iterations, we then estimated the proportion of the genome in which these 519 breakpoints would have been expected to occur, by chance alone, given a certain specified number of genomic regions available to harbour evolutionary breakpoints (Additional file 6). For these simulations, the human genome was partitioned into 10,000 regions, each 0.3 Mb in length (the average length of the observed breakpoint regions). Assuming a random breakage model for the entire genome, partitioned into 10,000 equal-sized genomic segments available to harbour breakpoint regions, the 519 evolutionary breakpoints would have been expected to occur in between 500 and 516 regions with 99% probability (Additional file 6). In other words, given random breakage, a maximum of 19/519 (3.7%) breakpoints might reasonably have been expected to co-locate by chance to the same regions at the 1% level of probability. In practice, however, we have noted that the 519 observed evolutionary breakpoints were confined to only 410 breakpoint regions. According to our simulations (presented in Additional file 6), this number of breakpoint regions would be expected if only 7–10% of the genome (i.e. 700–1000 of the 0.3 Mb regions) were available to harbour evolutionary breakpoints. Thus, according to our model-based simulations, the observation of 519 breakpoints being located within 410 out of 10,000 genomic regions is most plausible when the occurrence of breakpoints is confined to only 7–10% of the genome. Even if we were to assume that some 20% of the genome could harbour evolutionary breakpoints, the observed distribution has a <1% probability of occurring under the model of random breakage. We therefore feel confident in rejecting the null hypothesis that these breakage events occurred randomly. We instead conclude that they occurred preferentially within certain genomic regions.

Among the 519 breakpoints considered in the above mentioned simulation analysis were 27 breaks in synteny that occurred in the same genomic interval in both chicken and opossum, but not in mouse or cow. These breakpoints shared by chicken and opossum could however have been identical-by-descent and would thus have occurred only once in the eutherian common ancestor, not twice as we implicitly assumed in the previously described simulations. In order to avoid double counting of some breakpoints, we repeated the simulations, this time considering only the breakpoint regions in mouse (N = 132), cow (N = 143) and opossum (N = 89). A total of 41 breakpoint intervals were found to be shared by these species, whereas 323 breakpoint regions were unique to the species considered. During these simulations, the genome was subdivided into 10,000 bins, each of length 0.3 Mb (potential regions for a breakpoint), and the 323 mammalian breakpoints were distributed between these bins. The simulation experiments served to demonstrate that the breakpoint positions are incompatible with a random model of breakage. The expected number of breakpoint regions under this model was calculated to be 359.7; in none of the 100,000 simulation runs was such a low number of breakpoint intervals noted as that actually observed (N = 323; two-sided p-value approximates zero). When the model was relaxed to 2000 selected bins (special candidate regions for breakpoints), 342.6 unique breakpoints would have been expected (two-sided p = 0.00002). On the other hand, a model with 1000 bins, i.e. one using ~10% of the genome, appears to be compatible with the observed values: expected number of unique breakpoints = 322.3 (p = 0.92).

## Discussion

### Refining the structure of boreoeutherian ancestral chromosomes

Comparative genome maps, based on more than eighty species of eutherian mammal, have previously been generated by chromosome painting. Such analyses have revealed the pathways of mammalian genome evolution at the chromosomal level [6-8,10-12,54-57]. However, comparative chromosome painting is inadequate to the task of comparing the genomes of species which have been separated for more than 100 million years. This is due to the lower hybridization efficiency of probes consequent to increased sequence divergence. Thus, reports of successful hybridizations of eutherian probes onto marsupial chromosomes are confined to a single chromosome [58]. To overcome this limitation, comparative genome sequence analyses based on direct genome alignments have been performed with the aim of reconstructing precise ancestral gene orders [9,14-16]. However, models of ancestral eutherian genome organization constructed from such genome sequence alignments display considerable differences with respect to the assignment of ancestral syntenic groups, when compared to models derived from comparative chromosome painting data [12,19,20,59].

E-painting (electronic chromosome painting) [22] was introduced in order both to overcome the inherent limitations of comparative cytogenetic approaches and to reduce the complexity of direct whole genome sequence alignments. This *in silico *technique is based on the comparative mapping of orthologous genes and the identification of conserved syntenic segments of genes instead of comparative alignments of large sequence contigs containing intergenic sequences as well as genes. The advantage of E-painting over comparative genome sequence analysis is that the former reduces the complexity of genome alignments to easily manageable conserved syntenic segments comprising orthologous genes. Its limitation, however, is that it cannot be applied to the investigation of telomeric, centromeric or non-genic regions that could have nevertheless played an important role during karyotype evolution.

In the present study, E-painting was used to reinvestigate the previously proposed boreoeutherian protokaryotype [8,10,12,54]. The resulting model of the boreoeutherian genome (Figure 3) closely resembles those models previously derived by means of comparative chromosome painting. Indeed, our data derived from E-painting analysis not only confirmed all major syntenic segment associations proposed in previous studies [8-12] but also served to refine the model by accommodating short syntenic segments orthologous to portions of chromosomes *HSA*7, *HSA*10, *HSA*12 and *HSA*22 (Figure 3).

**Figure 4:**
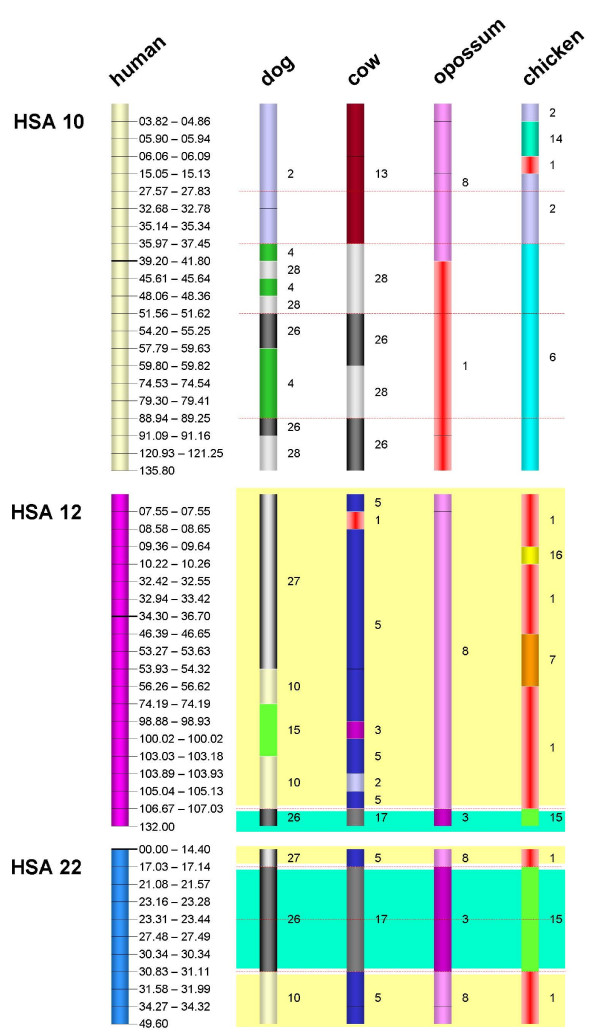
**E-painting results for chromosomes *HSA*10, *HSA*12 and *HSA*22**. The stippled red lines indicate regions of primate-specific breakpoints. Black lines within the ideograms represent the positions of breaks in synteny which were probably caused by inversions. Unique colour codes link the *HSA*12q distal segment (Mb 107.03–132.00) and the central 22q segment (Mb 17.14–30.83), representing the smallest eutherian chromosome [10,12] (12b-22b in Figure 2), as well as the segments 12pter-12q (Mb 0–106.67), 22q proximal (Mb 14.4–17.03), and 22q distal (Mb 31.11–49.60) representing a medium-sized eutherian chromosome (12a-22a in Figure 2). In dog and cow, the *HSA*10p orthologous segment (Mb 0–37.45) bears a colour code that is different from the *HSA*12 and *HSA*22 orthologues and hence does not provide any evidence for an evolutionary association. However, the shared synteny on opossum chromosome 8 confirms previously performed chromosome painting data [11,56,60], strongly suggesting common ancestral *HSA*10p/12pq/22q orthology. The E-painting data from the murids are not informative in this regard.

The improved definition of ancestral eutherian chromosomes by E-painting achieved in this study is particularly evident in the context of the evolution of chromosomes *HSA*12 and *HSA*22. A common feature of previously proposed protokaryotypes has been the presence of two different protochromosomes displaying associations of *HSA*12 and *HSA*22. As is evident from the colour-coded ideograms in Fig. 3, the larger protochromosome, 12p-q/22q, comprises an extended 12p-q segment stretching from *HSA*12pter to a point 106.67 Mb from 12q and includes the terminal segment of *HSA*22q (31.10 Mb toward 22qter). Further, we have identified a third proximal 2.7 Mb segment from *HSA*22q (14.4 Mb to 17.03 Mb) that bears the same colour code in all analyzed species (Figure 4) and which must therefore also form part of this large protochromosome. Additionally, the E-painting indicated that the ancestral chromosome orthologous to *HSA*10q should be extended by a 1.5 Mb-sized proximal portion of its p-arm (Figure 4). The existence of this extension was supported by both eutherian and chicken genome sequence data and indicates that the breakpoint is located in a region orthologous to 10p rather than within the centromere (Figure 4).

Importantly, E-painting using the opossum and chicken genomes indicated an *HSA*10p/12/22 association (Figure 4). These findings, taken together with recent comparative chromosome painting data supporting the 10p/12/22 association in the Afrotheria and in some Xenarthra [10,11,56,60] and carnivores [61], strongly corroborate an ancestral 10p/12/22 chromosome as part of the ancestral eutherian karyotype. Furthermore, this 10p/12/22 association is compatible with an ancestral eutherian chromosome number of 2n = 46 (Figure 3).

The extensive agreement between ancestral genome reconstructions based respectively upon comparative chromosome painting and E-painting is strongly supportive of the validity of the E-painting approach. Further, the E-painting analysis performed here has confirmed the previously proposed ancestral eutherian chromosome associations, 3/21, 4/8, 7/16, 10/12/22, 12/22, 16/19 and 14/15 [8-12], since all these associations are readily identifiable in the opossum genome. However, the 3/21 association in the opossum involves a different set of genes as compared to the 3/21 association in the eutherian species, thereby indicating the presence of additional rearrangements involving the corresponding chromosomal regions in marsupials.

Recent comparative chromosome painting studies performed with several afrotherian [10,55,60,62] and xenarthran species [11,56,63] have indicated that their karyotypes display a remarkable degree of similarity to the previously proposed ancestral boreoeutherian karyotype [12]. The chromosomal associations 1/19 and 5/21 seem, however, to be specific to afrotherians [55,56,62,64] with no xenarthran-specific chromosomal rearrangements having been identified as yet [11,56].

Our findings indicate that none of the afrotherian-specific rearrangements are evident in the opossum genome. This finding, together with the observation that the above mentioned ancestral eutherian chromosome associations are also present in the opossum, suggest that the ancestral boreoeutherian karyotype is very similar to the ancestral eutherian karyotype (see Additional file 3 for an overview of the phylogenetic relationships among the major placental groups, according to Wildman et al. [65]).

### Chromosomal distribution of evolutionary breakpoints

The comparative synteny analysis presented here has succeeded in defining evolutionary chromosomal breakpoints with a considerably higher degree of resolution than has previously been achieved. For example, the length of the median breakpoint interval in this study is only 120 kb (Table 2). Furthermore, the average length (290 kb) of the breakpoint intervals assigned here is about a quarter of that reported by Murphy et al. [9]. Ruiz-Herrera et al. [66], in a second related study, included data from Murphy et al. [9] but added further species with even less precisely defined breakpoint data. The present study has avoided the uncertainty inherent in matching up cytogenetic band information with genome sequence data. The assessment of the spatial correlation between evolutionary chromosomal breakpoints and DNA sequence features such as gene density, GC-content, segmental duplications and copy number variations (as well as cytogenetic features such as fragile sites and cancer-associated breakpoints), promises to yield new insights into mechanisms of chromosomal rearrangement whose relevance may well extend beyond the confines of evolution and into the sphere of genetic disease (and particularly tumorigenesis).

In this study, a total of 526 evolutionary breakpoint intervals were identified. Knowledge of their respective genomic positions then allowed us to address the question as to whether evolutionary breakpoints co-locate with cancer-associated breakpoints and/or common fragile sites, an issue which has been quite contentious over the last few years [23,67]. The original 'random breakage model' of Nadeau and Taylor [25] has been challenged by Pevzner and Tesler [68] who favour an alternative model in which at least some evolutionary breakpoint regions are prone to repeated breakage in the context of disease-related rearrangements. Inherent to the latter model is the prediction that evolutionary breaks will frequently overlap with fragile sites and cancer-associated breakpoints [9,66,69,70]. The precise mapping data presented here are not however compatible with such a physical overlap of breakpoints. When considering fragile sites, rare and common sites must be clearly distinguished [35]. Rare fragile sites are less frequent and, at the DNA sequence level, are associated with expanded repeats. In some cases, such sites are associated with a specific clinical phenotype [36]. By contrast, common fragile sites (numbering 89 according to Debacker and Kooy [36]) are observed in different mammalian species [71,72] and may be spatially associated with large active gene clusters [35]. In our analysis, we focussed exclusively on the 11 common fragile sites that have been well characterized at the DNA sequence level [35,38-49] but only two of these sites were found to exhibit partial overlap with an evolutionary breakpoint interval (N = 526) identified here (Table 4). We cannot however make any statement with respect to a potential overlap between the evolutionary breakpoints and those common fragile sites that are hitherto poorly mapped and remain uncharacterized at the DNA sequence level.

A second class of common chromosomal breakpoint is represented by those breakpoints associated with tumorigenesis. These cancer-related breakage events frequently generate fusion genes that are commonly characterized by gains of function [73]. To refine the DNA sequence positions of known cancer-associated breakpoints, we utilized the known sequence coordinates of 387 cancer-associated genes. These were then cross-compared with the 526 evolutionary breakpoint intervals identified in our analysis. However, no evidence was found for the known cancer-associated genes (and hence their associated breakpoint regions) being over-represented within regions of evolutionary chromosomal breakpoints.

A word of caution is appropriate here. Although it may eventually prove possible to identify unequivocally the positions of many evolutionary and cancer-associated breakpoints, there is no *a priori *reason to suppose that these breakpoints should occur in precisely the same locations. Indeed, there is every reason to believe that, even if we were to focus our attention upon those breakpoints which colocalize to the extended regions characterized by segmental duplication, these breakpoints would probably occur in heterogeneous locations with respect to the various genes present within the unstable regions. This is because, in order to come to clinical attention, somatic cancer-associated gene rearrangements must confer a growth advantage upon the affected cells or tissue, usually via gene deregulation or through the creation of a fusion gene. Evolutionary rearrangements (which must, by definition, be heritable and hence occur in germ cells) represent the other side of the coin: they could not have become fixed had they been disadvantageous to individuals of the species concerned. It follows that the rearrangements derived in these two quite different contexts (i.e. somatic/cancer-associated versus germ cell/evolutionary) are likely (i) to have affected the structure, function and expression of different genes in different ways, (ii) to have been subject to quite different 'selective pressures' in these different contexts and hence (iii) would have been most unlikely to have occurred in precisely the same genomic locations. In agreement with these predictions, a different regional distribution of cancer-associated and evolutionary breakpoints has been noted by Sankoff et al. [74] whilst Helmrich et al. [47] failed to detect any overlap between fragile sites and evolutionary breakpoints.

Our E-painting data do however provide some support for the postulate that evolutionary breakpoints have been 'reused', *sensu lato *[9]. Indeed, 7.6% of the identified evolutionary breakpoint intervals identified here contain two or more breakpoints. By computer simulation, we confirmed that the distribution of the 519 observed breakpoints into only 410 different genomic segments is best explained by non-random breakage with only ~7–10% of the genome harbouring evolutionary breakpoints. This proportion is somewhat lower than that previously reported (20%) for the 'reuse' of breakpoint regions [9] but this could be due to the higher resolution breakpoint mapping achieved here. Recently, breakpoint 'reuse' has also been noted in the case of a recurrent inversion on the eutherian X chromosome [75] and in a comparison of chicken chromosome *GGA*28 with orthologous syntenic segments in human, fish (*Fugu*), amphibian (*Xenopus*), opossum, dog and mouse [24]. Taken together, these findings are quite compatible with the fragile breakage model of chromosome evolution first proposed by Pevzner and Tesler [68] and sustained by the more recent analysis of Alekseyev and Pevzner [76].

Our data confirm and extend previous reports of associations between segmental duplications (SDs) with evolutionary rearrangements [77,78]. SDs comprise 4–5% of human autosomal euchromatin [50-52] whereas the primate lineage-specific breakpoint intervals comprise 0.86% of the euchromatin. This notwithstanding, some 78% of the evolutionary breakpoint intervals colocalize with known SDs whilst 76% coincide with regions of known copy number variation (Additional file 2). These proportions are significantly higher than those reported from comparative analyses of evolutionary breakpoints between the human and murine lineages [51,78]. This difference is probably due to the focus in the present analysis having been placed upon primate lineage-specific breakage.

Turning to the sites at which evolutionarily fixed chromosomal breaks have occurred, we have previously mapped at the DNA sequence level the breakpoints of eight inversions that serve to distinguish the human and chimpanzee karyotypes [79-81]. None of these rearrangements is as yet known to be associated with either the activation or inactivation of genes at or near the breakpoint sites. The present study indicates that, at least in the primate lineage, the evolutionary breakpoints are enriched for SDs whilst overlapping to a similar extent with sites of known copy number variants. This concurs with recent findings from comparative studies of syntenic disruptions between gibbon and human chromosomes [82,83]. Indeed, nearly half of all gibbon-human breaks in synteny occur within regions of segmental duplication in the human genome, thereby providing further evidence for the evolutionary plasticity of these regions which has clearly been responsible for promoting a significant proportion of the chromosomal breaks in primates [51].

Our analysis has revealed an even stronger correlation between high gene density and evolutionary fragility than that previously reported [9]. Although the evolutionary breakpoint regions identified here display about 3 to 4 times the transcript density of the euchromatic genome average (Table 7), it would seem rather unlikely that evolutionary breakpoints have frequently disrupted gene coding regions. Intriguingly, a study of chicken chromosome *GGA*28 [24] has revealed that evolutionary breakpoint regions, identified through the analysis of human-chicken synteny, are disproportionately located in regions with a high GC-content and high CpG island density rather than in gene-dense regions *per se*. Thus, it is tempting to speculate that at least some of these evolutionary breakpoints, particularly those occurring in gene-associated CpG-islands, could have contributed to functional changes in mammalian gene structure or expression [24].

## Conclusion

In summary, we have presented an approach that greatly reduces the complexity of comparative genome sequence analysis and which is capable of providing valuable insights into the dynamics of eutherian karyotype evolution. The gene synteny analysis data yielded high definition evolutionary breakpoint maps which have significantly improved the resolution of existing maps derived by chromosome painting [84]. Correlation analyses with similarly well mapped cancer-associated breakpoints and fragile sites however failed to provide any evidence for an association with evolutionary breakpoints. We nevertheless noted a higher than previously observed positive correlation of evolutionary breakpoints with gene density and also corroborated the reported association of segmental duplications with evolutionary breakpoints in the primate lineage. The ancestral eutherian genome, reconstructed through E-painting, displays a high degree of agreement with that derived from the much larger comparative cytogenetic dataset. The inclusion of a marsupial genome in this comparison, which has not hitherto been attempted, suggested that the ancestral boreoeutherian karyotype was probably very similar to the ancestral eutherian karyotype.

## Methods

### Gene synteny analysis

The synteny comparisons across different vertebrate species were carried out *in silico *by means of reciprocal BLAST 'best-hit' searches using the ENSEMBL database; . Only genomes with at least a 7-fold sequence coverage were included in the analysis (human, mouse, rat, cow, dog, chicken, opossum). Data mining for established protein coding genes was performed using the program BioMart (; ENSEMBL release 46). Orthologous gene location data were retrieved from the genomes of rat, mouse, dog, cow, opossum and chicken, and were arranged by reference to the human gene order (NCBI Build 36). For the purposes of this analysis, a syntenic segment was defined as consisting of a group of contiguous genes in humans as well as in the other species under investigation (mouse, rat or dog etc). We have included in these gene order comparisons all those human genes for which orthologues have been annotated in the genomes of mouse, rat, dog, cow, opossum and chicken. Only segments with three or more consecutive syntenic genes were considered in order to avoid annotation errors or the inclusion of pseudogenes and retrotransposed genes. To aid visualization, the syntenic segments were individually identified by differential colour coding according to the colour code given in Figure 2. Breakpoint intervals were defined by the last gene from the proximal syntenic segment and the first gene from the following more distal syntenic segment of the respective species (summarized in Additional file 2). Gene positions are given in Mb according to the human genome sequence . The data analysis was otherwise performed as previously described [22,34].

Gene density calculations were carried out using Stata software (StataCorp, College Station, TX) based on the transcriptome data presented by Versteeg et al. [53] with updates available through the Human Transcriptome Map .

The diploid chromosome numbers of the species investigated are: N = 40 in mouse; N = 42 in rat; N = 60 in cow; N = 78 in dog; N = 18 in opossum; N = 78 in chicken. The assembly of conserved syntenic segments into ancestral chromosomes was used to model the ancestral boreoeutherian karyotype with a chromosome number of 2n = 46.

### Bovine genome versions

At the time of writing, the bovine genome sequence remains unpublished although a near complete version (B_tau3.1) was made available to us for the purposes of this study B_tau3.1 . B_tau3.1 has recently been replaced by the latest version B_tau4.0. The only major differences between the two versions of the bovine genome sequence resulted from scaffolds being misplaced within chromosomes *BTA*6, 19 and 29, respectively. These errors could however only account for the misclassification of intrachromosomal rearrangement breakpoints. Our synteny comparisons were, by contrast, largely based on the identification of interchromosomal rearrangements (syntenic genes in humans being located on two different chromosomes in the species under investigation). Nevertheless, re-examination of our data allowed us to conclude that our original results were not affected in any way by the occasional intrachromosomal misplacement of scaffolds on the *BTA *chromosomes in version B_tau3.1. All six intrachromosomal breakpoints (involving *BTA *chromosomes 6, 19 and 29) were found to coincide with breakpoints identified in other species (Additional file 1). Indeed, four of these 6 intrachromosomal breakpoints coincided with breakpoints in two or more additional species. It therefore follows that the removal of these B_tau3.1-derived 'breakpoints' from our analysis would not have resulted in any reduction in the overall breakpoint number.

### Assessment of overlap between evolutionary breakpoints and common fragile sites

The χ^2^-goodness-of-fit (exact version implemented in SAS) was applied to test whether the overlap between autosomal fragile sites and evolutionary breakpoint intervals is non-random. The genomic region covered by 11 selected fragile sites is 34.6 Mb, as summarized in Table 4, amounting to 1.12% of the autosomal genome (assuming it to be 3093 Mb). Since the average extension of a breakpoint interval is 0.3 Mb, it is on average sufficient for an overlap that the midpoint of a breakpoint interval lies within the borders of a fragile site ± 0.15 Mb, an area which amounts to 34.6+11 × 0.3 = 37.9 Mb. Thus, under a random model, ~1.23% (37.9/3093) of the 526 observed breakpoints intervals would be predicted to overlap with a fragile site. Since only 2/526 breakpoints (0.38%) were found to display an overlap with a fragile site (p = 0.11), there was no evidence for significant co-location.

### Simulation experiments

To assess whether the positions of the breakpoints identified in this study would fit best with a model of random or non-random chromosomal breakage during vertebrate karyotype evolution, 100,000 simulation experiments were performed. Depending upon the number of genomic regions of length 0.3 Mb available for evolutionary breakpoints, the expected number of different breakpoint regions assumed to harbour a total of 519 observed breakpoints (N = 132 in mouse, 143 in cow, 89 in opossum and 155 in chicken) was estimated under a model of random breakpoint selection in each species. The deduced relationship between the number of genomic segments available for chromosomal breakage and the expected and observed numbers of genomic segments used by 519 breakpoints has been graphically depicted (Additional file 6). Additionally, the '99%-probability intervals' were determined to provide an indication of the ranges over which the different breakpoint regions are situated with a probability of 99%. The expected numbers of genomic segments were then directly compared with the observed number of 410 regions actually used. Thus, for example, if 1000 segments (corresponding to ~10% of the genome) were available to harbour evolutionary breakpoints, some 427 would have been expected to be used by 519 breakpoints. The probability that <409 or >445 segments would contain a breakpoint was calculated to be only ~1%.

## Abbreviations

MYA: million years ago; Mb: megabase.

## Competing interests

The authors declare that they have no competing interests.

## Authors' contributions

HH and HKS conceived and designed the experiments. CK and MK performed the experiments. HKS, LF, HH and DNC analyzed the data and wrote the paper. JH performed the statistical analysis. All authors read and approved the final manuscript.

## Supplementary Material

Additional file 1 **Summary of E-painting results for human chromosomes 1–22. **The conversion of the syntenic segment associations into colour-coded ideograms rendered the conserved syntenic segments (and at the same time, the breakpoint intervals) readily identifiable The human chromosome coordinates of the breakpoint intervals are given to the right of the human ideogram in Mb. The chromosome numbers of the orthologous segments in the analyzed species are indicated to the right of the conserved segments. Chromosomal breakpoints have been evenly spaced in order to facilitate visualization of the conserved syntenic segments. The resulting ideograms of the chromosomes and conserved segments are therefore not drawn to scale. The centromeric region is indicated by a black horizontal bar. The stippled red lines indicate breaks present in all analyzed vertebrate genomes which may therefore be attributed to rearrangements in the primate lineage. Click here for file

Additional file 2 **Summary of the evolutionary breaks for all human autosomal chromosomes**. The breakpoint intervals are demarcated by the last gene from the proximal segment of conserved synteny and the first gene of the distal segment of conserved synteny. For some breaks, a duplication of the gene at the breakpoint was noted (duplicated genes). The breaks specific to the primate lineage are marked in yellow. For these primate-specific breaks, the links to copy number variants (CNV) and segmental duplications (SD) contained within the break intervals have been added. In the last column, the cancer-associated genes which flank evolutionary breaks (these genes are included within the respective segments of common synteny) and the known cancer-associated genes localized within the break intervals (see Table 5) are given. Click here for file

Additional file 3 **Phylogenetic relationships among the species investigated in this study and the armadillo and the elephant not analysed here (marked with an asterisk) according to Wildman et al. **[65]. Scheme to indicate phylogenetic relationships between clades. Click here for file

Additional file 4 **Average transcript density of all evolutionary breakpoint intervals on human chromosomes**. Table indicating the average transcript density of all evolutionary breakpoint intervals. Click here for file

Additional file 5 **Average transcript density of evolutionary breakpoint intervals mapped to regions under 40 kb (n = 144)**. Table indicating the average transcript density of evolutionary breakpoints. Click here for file

Additional file 6 **Results of 100,000 simulation experiments designed to investigate the question of random or non-random breakage during mammalian karyotype evolution**. Table indicating the results of 100,000 simulation experiments designed to investigate the question of random or non-random breakage during mammalian karyotype evolution. Click here for file
