# Supplementary material for: Gene synteny comparisons between different vertebrates provide new insights into breakage and fusion events during mammalian karyotype evolution
Source: BMC Evol Biol. 2009 Apr 24;9:84. doi: 10.1186/1471-2148-9-84 (PMC2681463; doi:10.1186/1471-2148-9-84)
Supplement: Additional file 1 — Summary of E-painting results for human chromosomes 1–22. The conversion of the syntenic segment associations into colour-coded ideograms rendered the conserved syntenic segments (and at the same time, the breakpoint intervals) readily identifiable The human chromosome coordinates of the breakpoint intervals are given to the right of the human ideogram in Mb. The chromosome numbers of the orthologous segments in the analyzed species are indicated to the right of the conserved segments. Chromosomal breakpoints have been evenly spaced in order to facilitate visualization of the conserved syntenic segments. The resulting ideograms of the chromosomes and conserved segments are therefore not drawn to scale. The centromeric region is indicated by a black horizontal bar. The stippled red lines indicate breaks present in all analyzed vertebrate genomes which may therefore be attributed to rearrangements in the primate lineage. [file 1471-2148-9-84-S1.ppt]

## Slide 1
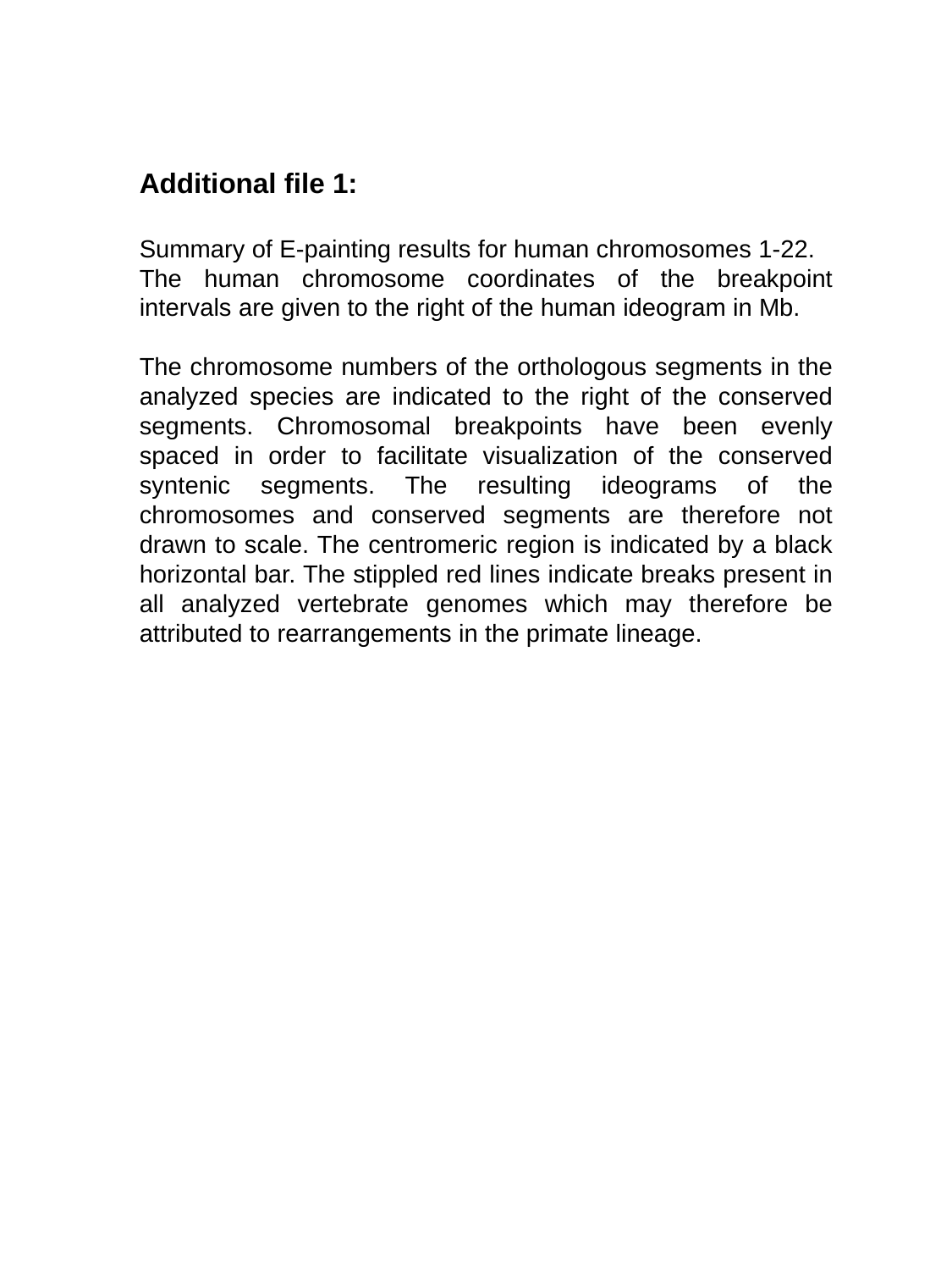

Additional file 1:
Summary of E-painting results for human chromosomes 1-22.
The human chromosome coordinates of the breakpoint intervals are given to the right of the human ideogram in Mb.
The chromosome numbers of the orthologous segments in the analyzed species are indicated to the right of the conserved segments. Chromosomal breakpoints have been evenly spaced in order to facilitate visualization of the conserved syntenic segments. The resulting ideograms of the chromosomes and conserved segments are therefore not drawn to scale. The centromeric region is indicated by a black horizontal bar. The stippled red lines indicate breaks present in all analyzed vertebrate genomes which may therefore be attributed to rearrangements in the primate lineage.

## Slide 2
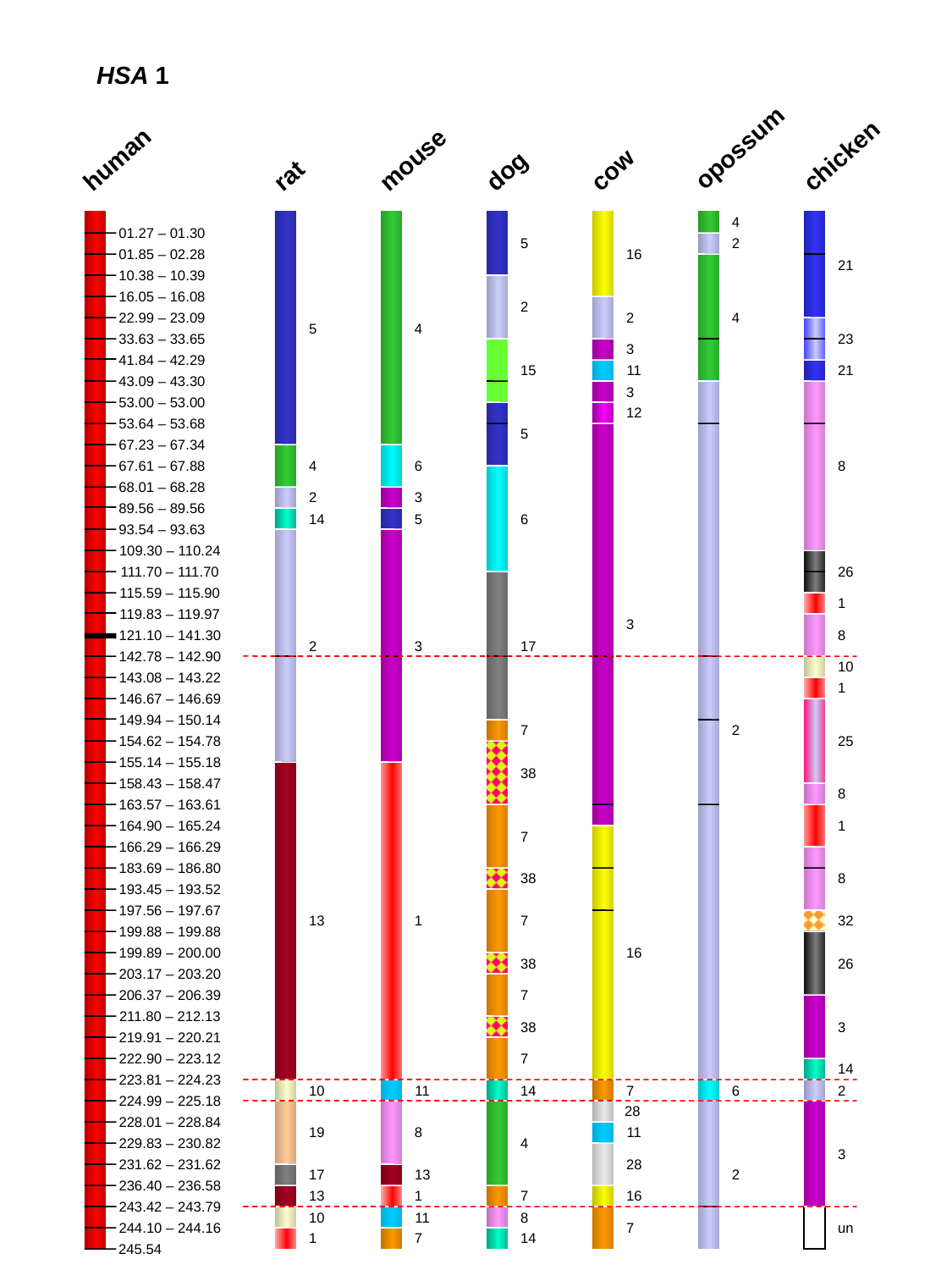

HSA 1
opossum
chicken
human
mouse
cow
dog
rat
4
01.27 – 01.30
5
2
01.85 – 02.28
16
21
10.38 – 10.39
16.05 – 16.08
2
22.99 – 23.09
2
4
5
4
33.63 – 33.65
23
3
41.84 – 42.29
15
11
21
43.09 – 43.30
3
53.00 – 53.00
12
53.64 – 53.68
5
67.23 – 67.34
67.61 – 67.88
4
6
8
68.01 – 68.28
2
3
89.56 – 89.56
14
5
6
93.54 – 93.63
109.30 – 110.24
111.70 – 111.70
26
115.59 – 115.90
1
119.83 – 119.97
3
121.10 – 141.30
8
2
3
17
142.78 – 142.90
10
143.08 – 143.22
1
146.67 – 146.69
149.94 – 150.14
7
2
154.62 – 154.78
25
155.14 – 155.18
38
158.43 – 158.47
8
163.57 – 163.61
164.90 – 165.24
1
7
166.29 – 166.29
183.69 – 186.80
38
8
193.45 – 193.52
197.56 – 197.67
13
1
7
32
199.88 – 199.88
199.89 – 200.00
16
38
26
203.17 – 203.20
206.37 – 206.39
7
211.80 – 212.13
38
3
219.91 – 220.21
222.90 – 223.12
7
14
223.81 – 224.23
10
11
14
7
6
2
224.99 – 225.18
28
228.01 – 228.84
19
8
11
229.83 – 230.82
4
3
231.62 – 231.62
28
17
13
2
236.40 – 236.58
13
1
7
16
243.42 – 243.79
10
11
8
244.10 – 244.16
7
un
1
7
14
245.54

## Slide 3
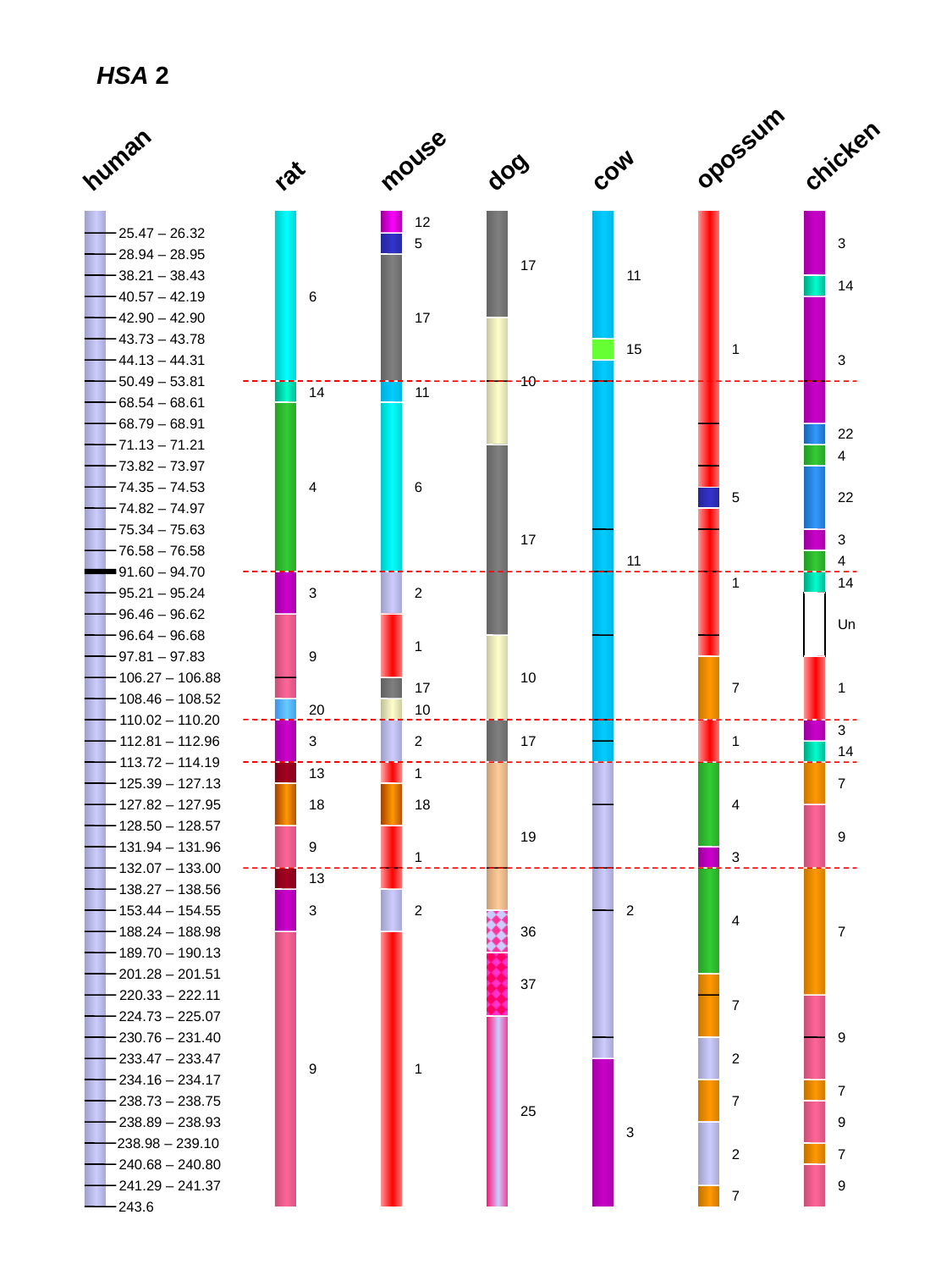

HSA 2
opossum
chicken
human
mouse
cow
dog
rat
12
25.47 – 26.32
5
3
28.94 – 28.95
17
38.21 – 38.43
11
14
40.57 – 42.19
6
42.90 – 42.90
17
43.73 – 43.78
15
1
44.13 – 44.31
3
50.49 – 53.81
10
14
11
68.54 – 68.61
68.79 – 68.91
22
71.13 – 71.21
4
73.82 – 73.97
74.35 – 74.53
4
6
5
22
74.82 – 74.97
75.34 – 75.63
17
3
76.58 – 76.58
11
4
91.60 – 94.70
1
14
95.21 – 95.24
3
2
96.46 – 96.62
Un
96.64 – 96.68
1
97.81 – 97.83
9
106.27 – 106.88
10
17
7
1
108.46 – 108.52
20
10
110.02 – 110.20
3
112.81 – 112.96
3
2
17
1
14
113.72 – 114.19
13
1
125.39 – 127.13
7
127.82 – 127.95
18
18
4
128.50 – 128.57
19
9
131.94 – 131.96
9
1
3
132.07 – 133.00
13
138.27 – 138.56
153.44 – 154.55
3
2
2
4
188.24 – 188.98
36
7
189.70 – 190.13
201.28 – 201.51
37
220.33 – 222.11
7
224.73 – 225.07
230.76 – 231.40
9
233.47 – 233.47
2
9
1
234.16 – 234.17
7
238.73 – 238.75
7
25
238.89 – 238.93
9
3
238.98 – 239.10
2
7
240.68 – 240.80
241.29 – 241.37
9
7
243.6

## Slide 4
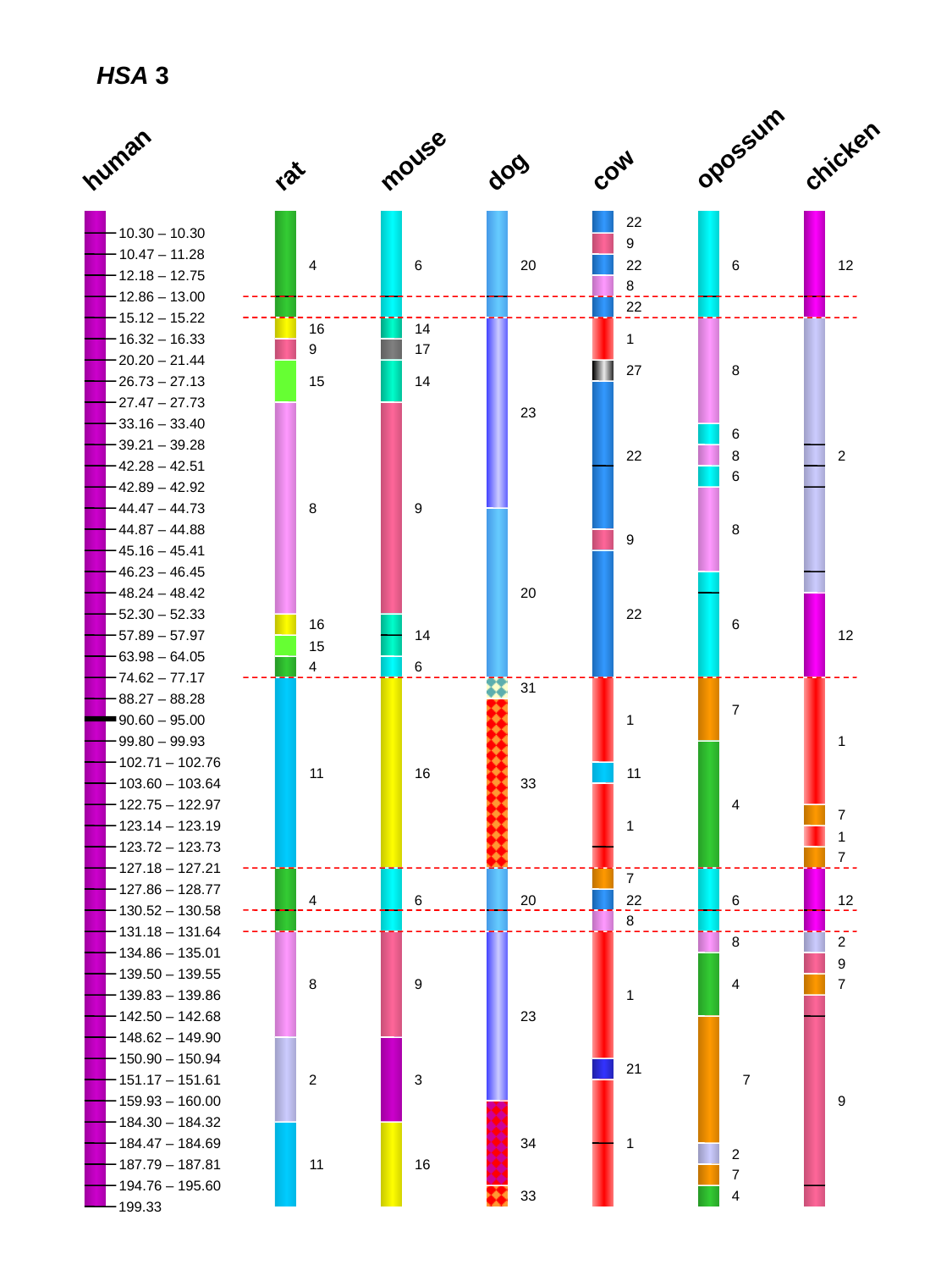

HSA 3
opossum
chicken
human
mouse
cow
dog
rat
22
10.30 – 10.30
9
10.47 – 11.28
4
6
20
22
6
12
12.18 – 12.75
8
12.86 – 13.00
22
15.12 – 15.22
16
14
16.32 – 16.33
1
9
17
20.20 – 21.44
27
8
26.73 – 27.13
15
14
27.47 – 27.73
23
33.16 – 33.40
6
39.21 – 39.28
22
8
2
42.28 – 42.51
6
42.89 – 42.92
44.47 – 44.73
8
9
44.87 – 44.88
8
9
45.16 – 45.41
46.23 – 46.45
48.24 – 48.42
20
52.30 – 52.33
22
16
6
57.89 – 57.97
14
12
15
63.98 – 64.05
4
6
74.62 – 77.17
31
88.27 – 88.28
7
90.60 – 95.00
1
99.80 – 99.93
1
102.71 – 102.76
11
16
11
103.60 – 103.64
33
122.75 – 122.97
4
7
123.14 – 123.19
1
1
123.72 – 123.73
7
127.18 – 127.21
7
127.86 – 128.77
4
6
20
22
6
12
130.52 – 130.58
8
131.18 – 131.64
8
2
134.86 – 135.01
9
139.50 – 139.55
8
9
4
7
139.83 – 139.86
1
142.50 – 142.68
23
148.62 – 149.90
150.90 – 150.94
21
151.17 – 151.61
2
3
7
159.93 – 160.00
9
184.30 – 184.32
184.47 – 184.69
34
1
2
187.79 – 187.81
11
16
7
194.76 – 195.60
33
4
199.33

## Slide 5
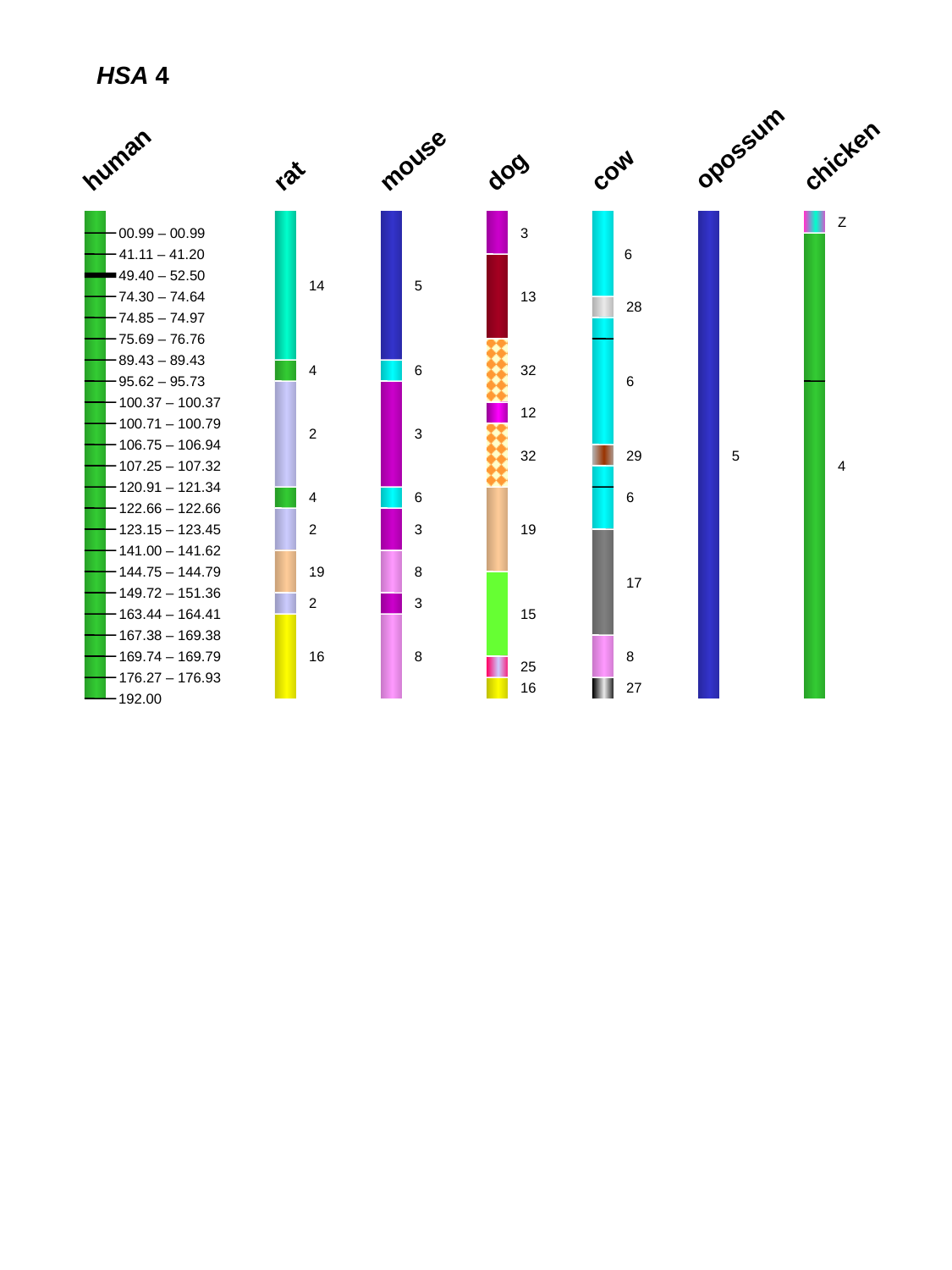

HSA 4
opossum
chicken
human
mouse
cow
dog
rat
Z
00.99 – 00.99
3
41.11 – 41.20
6
49.40 – 52.50
14
5
74.30 – 74.64
13
28
74.85 – 74.97
75.69 – 76.76
89.43 – 89.43
4
6
32
95.62 – 95.73
6
100.37 – 100.37
12
100.71 – 100.79
2
3
106.75 – 106.94
32
29
5
107.25 – 107.32
4
120.91 – 121.34
4
6
6
122.66 – 122.66
123.15 – 123.45
2
3
19
141.00 – 141.62
144.75 – 144.79
19
8
17
149.72 – 151.36
2
3
163.44 – 164.41
15
167.38 – 169.38
169.74 – 169.79
16
8
8
25
176.27 – 176.93
16
27
192.00

## Slide 6
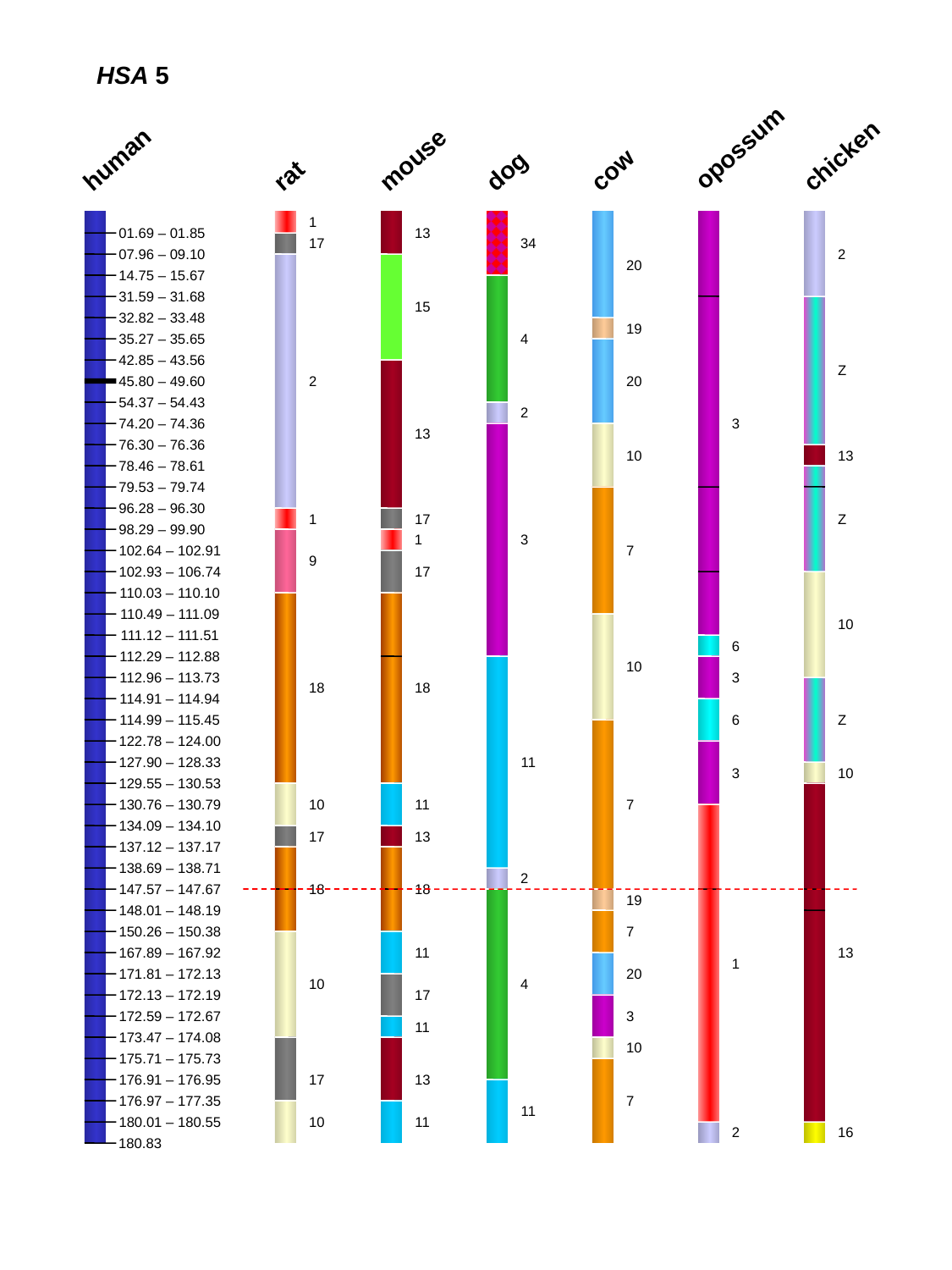

HSA 5
opossum
chicken
human
mouse
cow
dog
rat
1
01.69 – 01.85
13
17
34
07.96 – 09.10
2
20
14.75 – 15.67
31.59 – 31.68
15
32.82 – 33.48
19
35.27 – 35.65
4
42.85 – 43.56
Z
45.80 – 49.60
2
20
54.37 – 54.43
2
74.20 – 74.36
3
13
76.30 – 76.36
10
13
78.46 – 78.61
79.53 – 79.74
96.28 – 96.30
1
17
Z
98.29 – 99.90
1
3
102.64 – 102.91
7
9
102.93 – 106.74
17
110.03 – 110.10
110.49 – 111.09
10
111.12 – 111.51
6
112.29 – 112.88
10
112.96 – 113.73
3
18
18
114.91 – 114.94
114.99 – 115.45
6
Z
122.78 – 124.00
127.90 – 128.33
11
3
10
129.55 – 130.53
130.76 – 130.79
10
11
7
134.09 – 134.10
17
13
137.12 – 137.17
138.69 – 138.71
2
147.57 – 147.67
18
18
19
148.01 – 148.19
150.26 – 150.38
7
167.89 – 167.92
11
13
1
171.81 – 172.13
20
10
4
172.13 – 172.19
17
172.59 – 172.67
3
11
173.47 – 174.08
10
175.71 – 175.73
176.91 – 176.95
17
13
176.97 – 177.35
7
11
180.01 – 180.55
10
11
2
16
180.83

## Slide 7
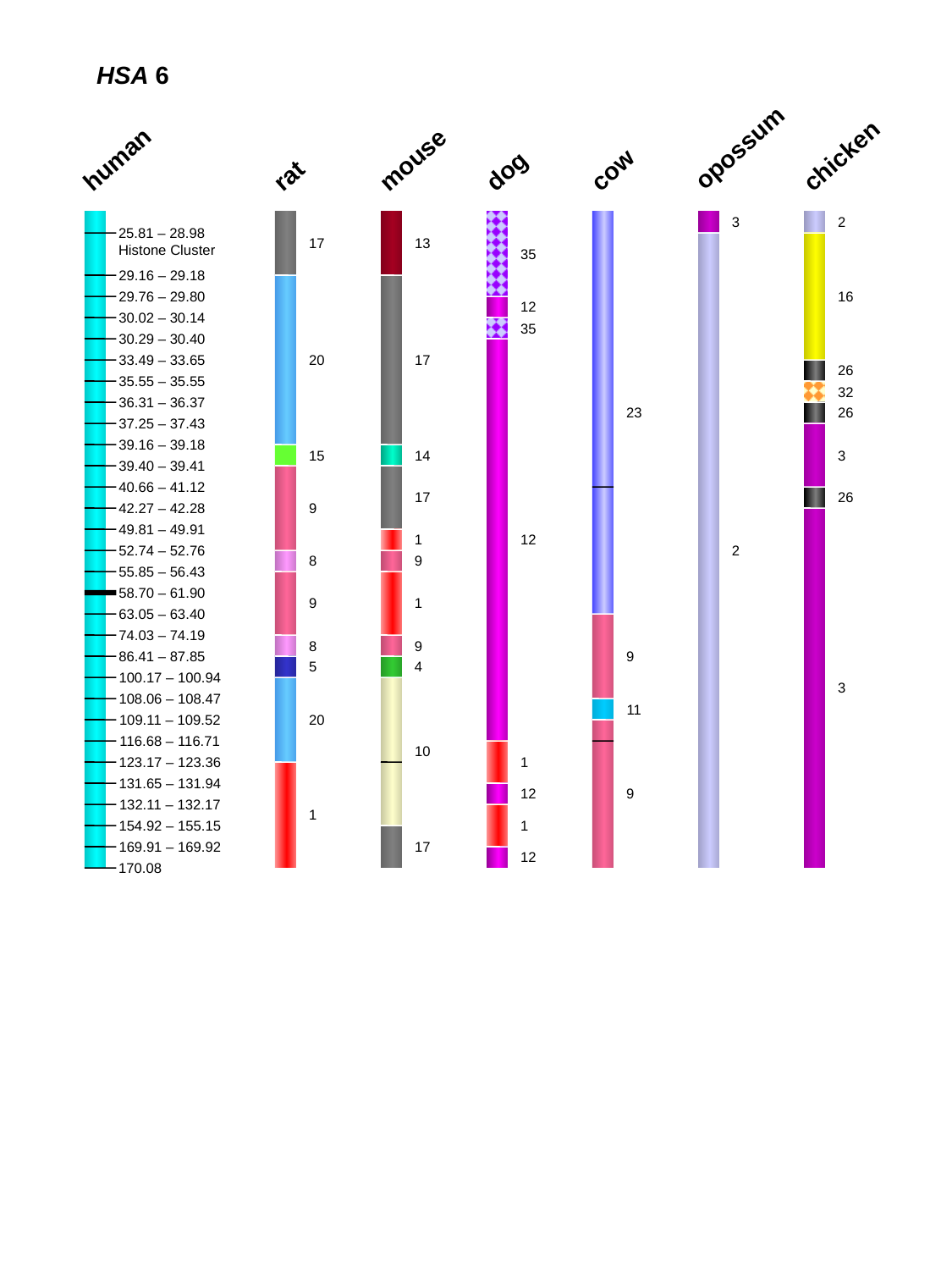

HSA 6
opossum
chicken
human
mouse
cow
dog
rat
3
2
25.81 – 28.98
Histone Cluster
17
13
35
29.16 – 29.18
29.76 – 29.80
16
12
30.02 – 30.14
35
30.29 – 30.40
33.49 – 33.65
20
17
26
35.55 – 35.55
32
36.31 – 36.37
23
26
37.25 – 37.43
39.16 – 39.18
15
14
3
39.40 – 39.41
40.66 – 41.12
17
26
42.27 – 42.28
9
49.81 – 49.91
1
12
52.74 – 52.76
2
8
9
55.85 – 56.43
58.70 – 61.90
9
1
63.05 – 63.40
74.03 – 74.19
8
9
86.41 – 87.85
9
5
4
100.17 – 100.94
3
108.06 – 108.47
11
109.11 – 109.52
20
116.68 – 116.71
10
123.17 – 123.36
1
131.65 – 131.94
12
9
132.11 – 132.17
1
154.92 – 155.15
1
169.91 – 169.92
17
12
170.08

## Slide 8
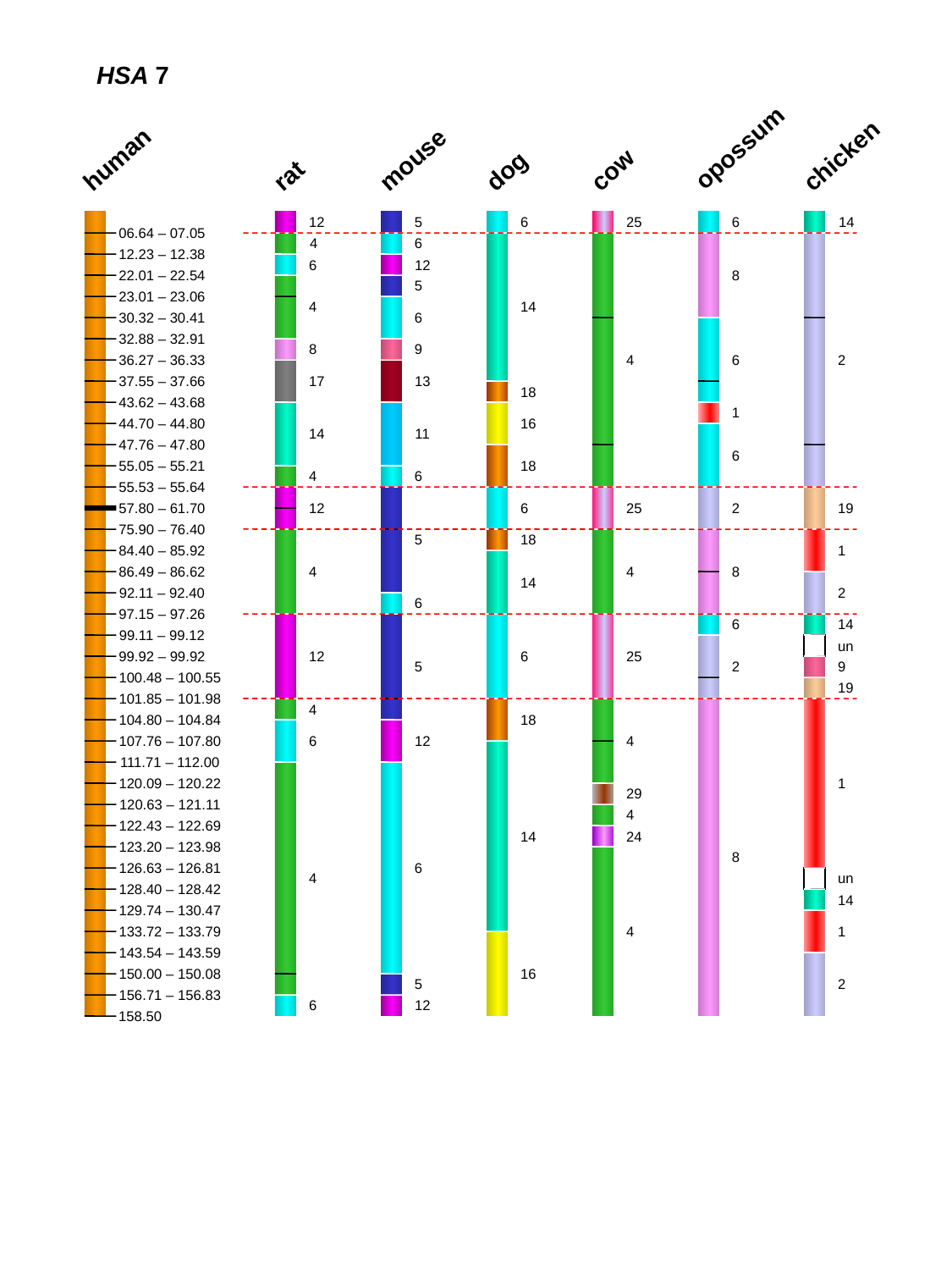

HSA 7
opossum
chicken
human
mouse
cow
dog
rat
12
5
6
25
6
14
06.64 – 07.05
4
6
12.23 – 12.38
6
12
22.01 – 22.54
8
5
23.01 – 23.06
4
14
30.32 – 30.41
6
32.88 – 32.91
8
9
36.27 – 36.33
4
6
2
37.55 – 37.66
17
13
18
43.62 – 43.68
1
44.70 – 44.80
16
14
11
47.76 – 47.80
6
55.05 – 55.21
18
4
6
55.53 – 55.64
57.80 – 61.70
12
6
25
2
19
75.90 – 76.40
5
18
84.40 – 85.92
1
86.49 – 86.62
4
4
8
14
92.11 – 92.40
2
6
97.15 – 97.26
6
14
99.11 – 99.12
un
99.92 – 99.92
12
6
25
5
2
9
100.48 – 100.55
19
101.85 – 101.98
4
104.80 – 104.84
18
107.76 – 107.80
6
12
4
111.71 – 112.00
120.09 – 120.22
1
29
120.63 – 121.11
4
122.43 – 122.69
14
24
123.20 – 123.98
8
126.63 – 126.81
6
4
un
128.40 – 128.42
14
129.74 – 130.47
133.72 – 133.79
4
1
143.54 – 143.59
150.00 – 150.08
16
5
2
156.71 – 156.83
6
12
158.50

## Slide 9
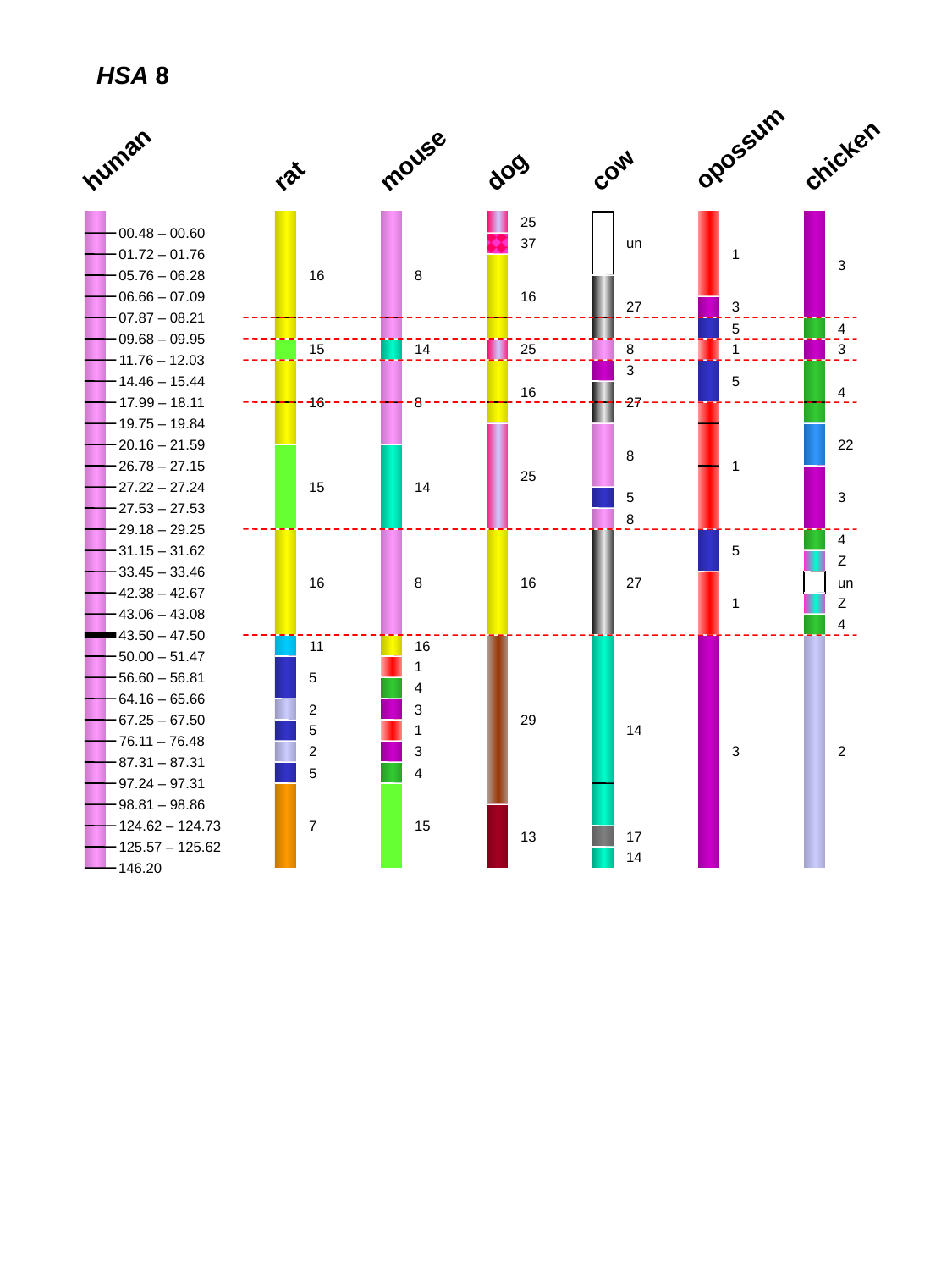

HSA 8
opossum
chicken
human
mouse
cow
dog
rat
25
00.48 – 00.60
37
un
01.72 – 01.76
1
3
05.76 – 06.28
16
8
06.66 – 07.09
16
27
3
07.87 – 08.21
5
4
09.68 – 09.95
15
14
25
8
1
3
11.76 – 12.03
3
14.46 – 15.44
5
16
4
17.99 – 18.11
16
8
27
19.75 – 19.84
20.16 – 21.59
22
8
26.78 – 27.15
1
25
27.22 – 27.24
15
14
5
3
27.53 – 27.53
8
29.18 – 29.25
4
31.15 – 31.62
5
Z
33.45 – 33.46
16
8
16
27
un
42.38 – 42.67
1
Z
43.06 – 43.08
4
43.50 – 47.50
11
16
50.00 – 51.47
1
56.60 – 56.81
5
4
64.16 – 65.66
2
3
67.25 – 67.50
29
5
1
14
76.11 – 76.48
2
3
3
2
87.31 – 87.31
5
4
97.24 – 97.31
98.81 – 98.86
124.62 – 124.73
7
15
13
17
125.57 – 125.62
14
146.20

## Slide 10
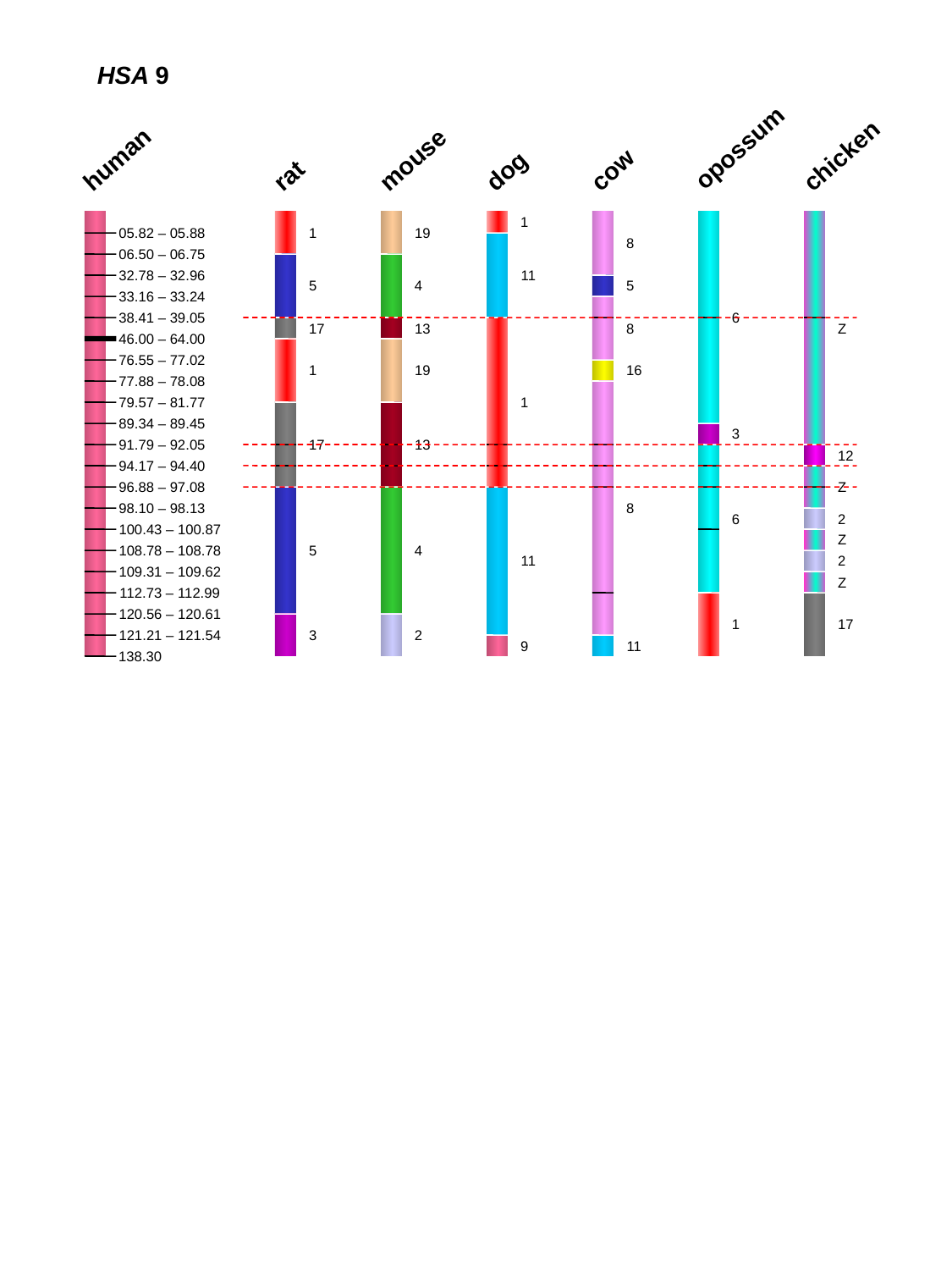

HSA 9
opossum
chicken
human
mouse
cow
dog
rat
1
05.82 – 05.88
1
19
8
06.50 – 06.75
32.78 – 32.96
11
5
4
5
33.16 – 33.24
38.41 – 39.05
6
17
13
8
Z
46.00 – 64.00
76.55 – 77.02
1
19
16
77.88 – 78.08
79.57 – 81.77
1
89.34 – 89.45
3
91.79 – 92.05
17
13
12
94.17 – 94.40
96.88 – 97.08
Z
98.10 – 98.13
8
6
2
100.43 – 100.87
Z
108.78 – 108.78
5
4
11
2
109.31 – 109.62
Z
112.73 – 112.99
120.56 – 120.61
1
17
121.21 – 121.54
3
2
9
11
138.30

## Slide 11
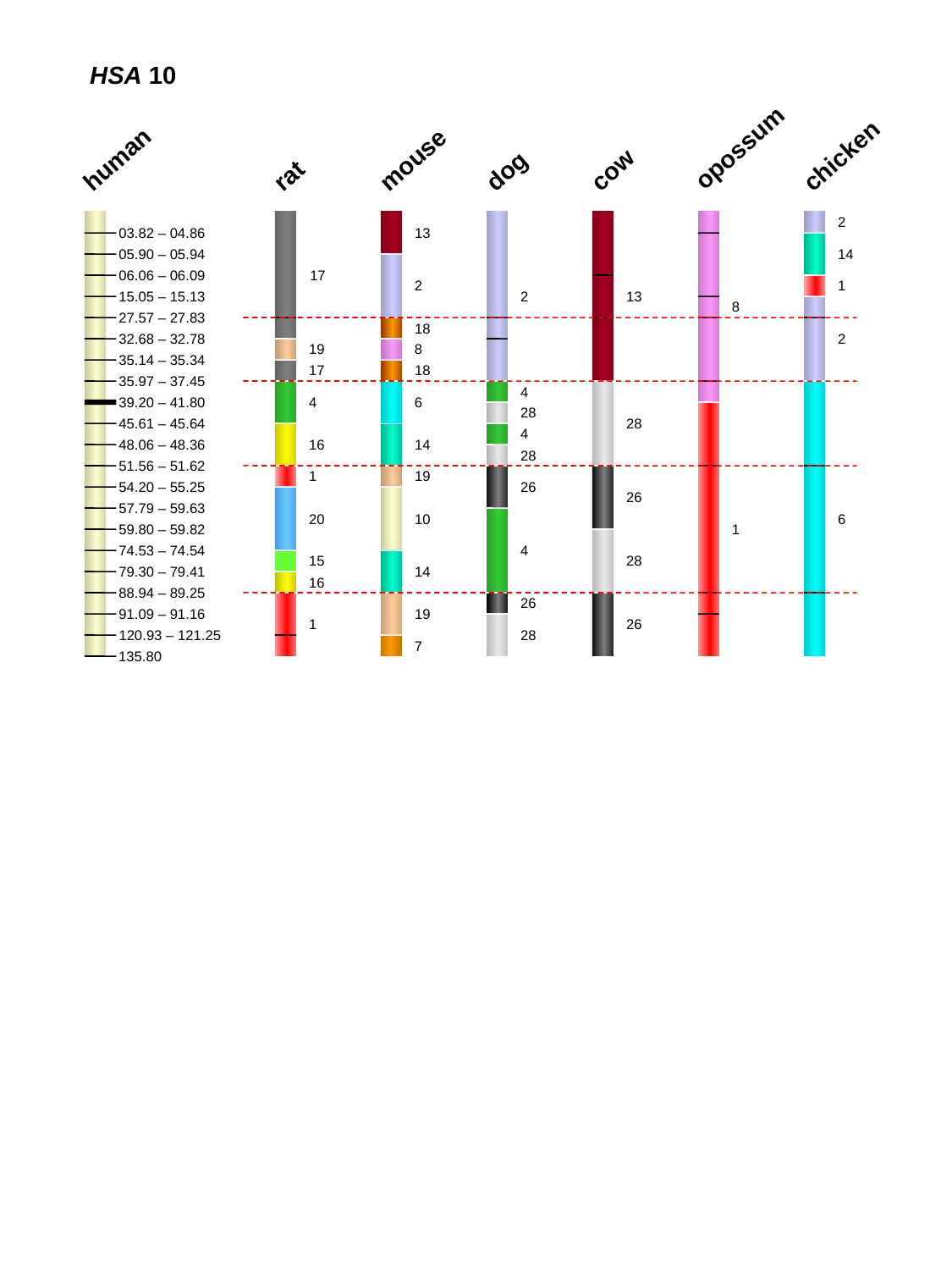

HSA 10
opossum
chicken
human
mouse
cow
dog
rat
2
03.82 – 04.86
13
05.90 – 05.94
14
06.06 – 06.09
17
2
1
15.05 – 15.13
2
13
8
27.57 – 27.83
18
32.68 – 32.78
2
19
8
35.14 – 35.34
17
18
35.97 – 37.45
4
39.20 – 41.80
4
6
28
45.61 – 45.64
28
4
48.06 – 48.36
16
14
28
51.56 – 51.62
1
19
54.20 – 55.25
26
26
57.79 – 59.63
20
10
6
59.80 – 59.82
1
74.53 – 74.54
4
15
28
79.30 – 79.41
14
16
88.94 – 89.25
26
91.09 – 91.16
19
1
26
120.93 – 121.25
28
7
135.80

## Slide 12
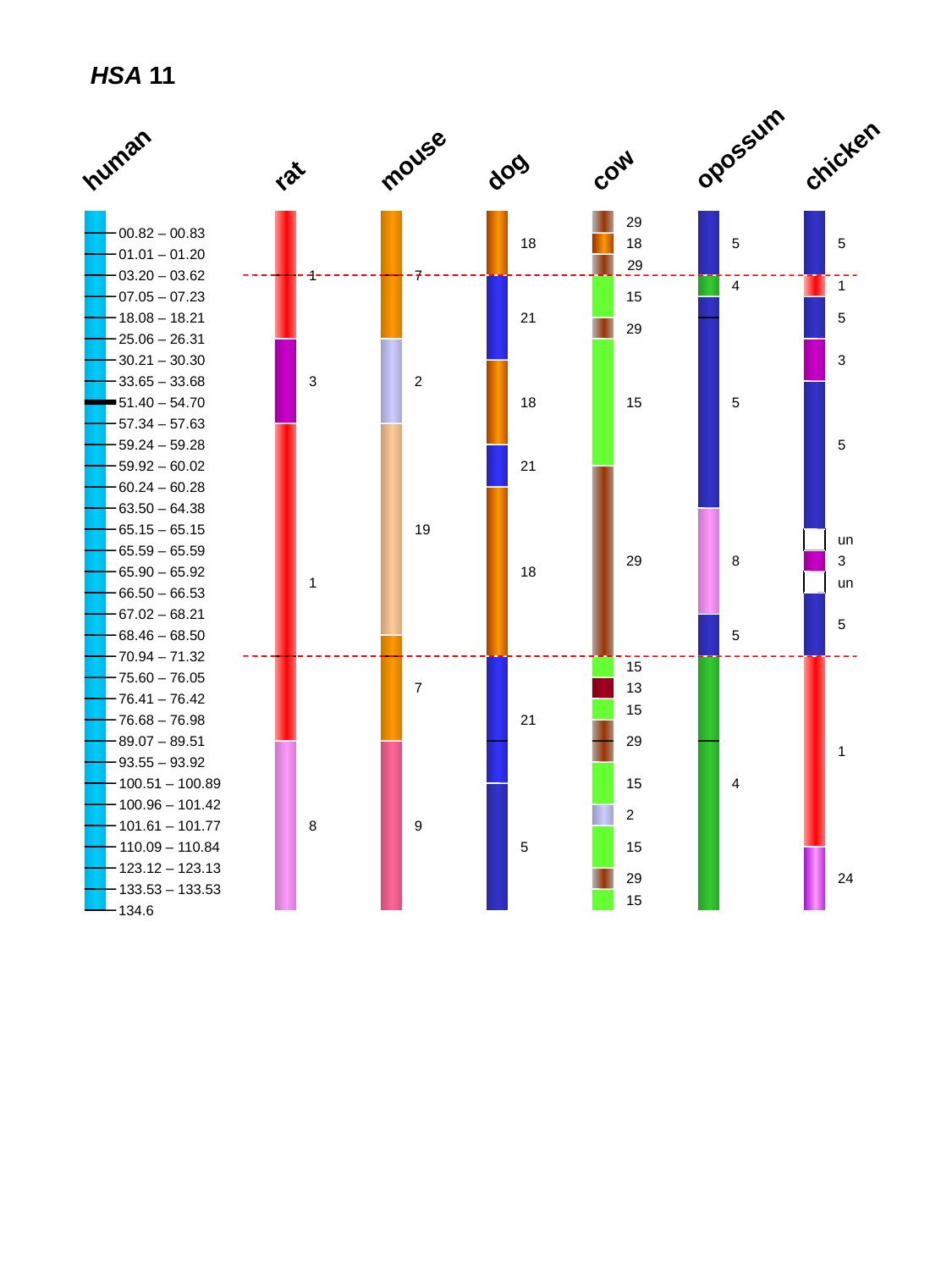

HSA 11
opossum
chicken
human
mouse
cow
dog
rat
29
00.82 – 00.83
18
18
5
5
01.01 – 01.20
29
03.20 – 03.62
1
7
4
1
07.05 – 07.23
15
18.08 – 18.21
21
5
29
25.06 – 26.31
30.21 – 30.30
3
33.65 – 33.68
3
2
51.40 – 54.70
18
15
5
57.34 – 57.63
59.24 – 59.28
5
59.92 – 60.02
21
60.24 – 60.28
63.50 – 64.38
65.15 – 65.15
19
un
65.59 – 65.59
29
8
3
65.90 – 65.92
18
1
un
66.50 – 66.53
67.02 – 68.21
5
68.46 – 68.50
5
70.94 – 71.32
15
75.60 – 76.05
7
13
76.41 – 76.42
15
76.68 – 76.98
21
89.07 – 89.51
29
1
93.55 – 93.92
100.51 – 100.89
15
4
100.96 – 101.42
2
101.61 – 101.77
8
9
110.09 – 110.84
5
15
123.12 – 123.13
29
24
133.53 – 133.53
15
134.6

## Slide 13
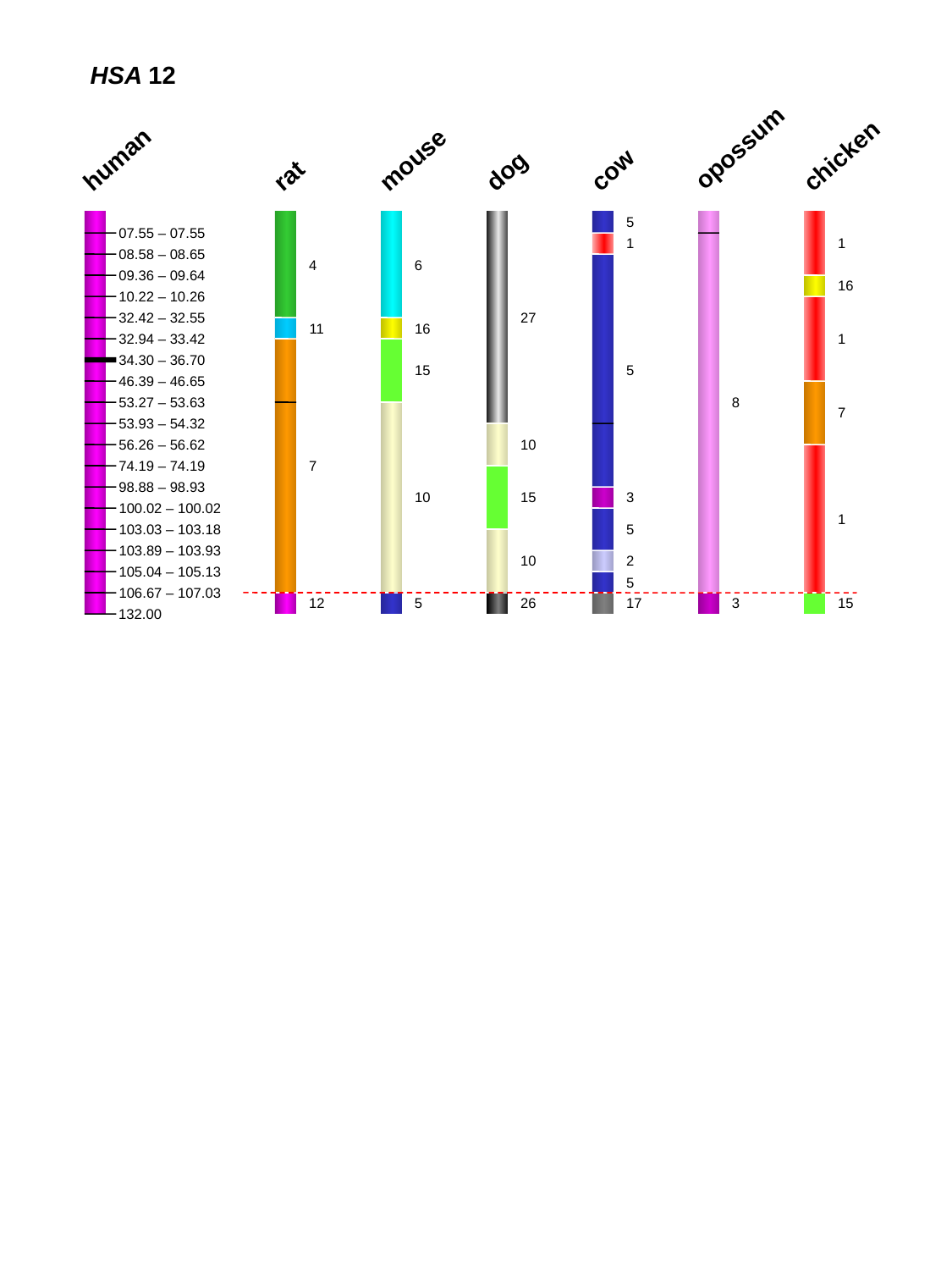

HSA 12
opossum
chicken
human
mouse
cow
dog
rat
5
07.55 – 07.55
1
1
08.58 – 08.65
4
6
09.36 – 09.64
16
10.22 – 10.26
32.42 – 32.55
27
11
16
32.94 – 33.42
1
34.30 – 36.70
15
5
46.39 – 46.65
53.27 – 53.63
8
7
53.93 – 54.32
56.26 – 56.62
10
74.19 – 74.19
7
98.88 – 98.93
10
15
3
100.02 – 100.02
1
103.03 – 103.18
5
103.89 – 103.93
10
2
105.04 – 105.13
5
106.67 – 107.03
12
5
26
17
3
15
132.00

## Slide 14
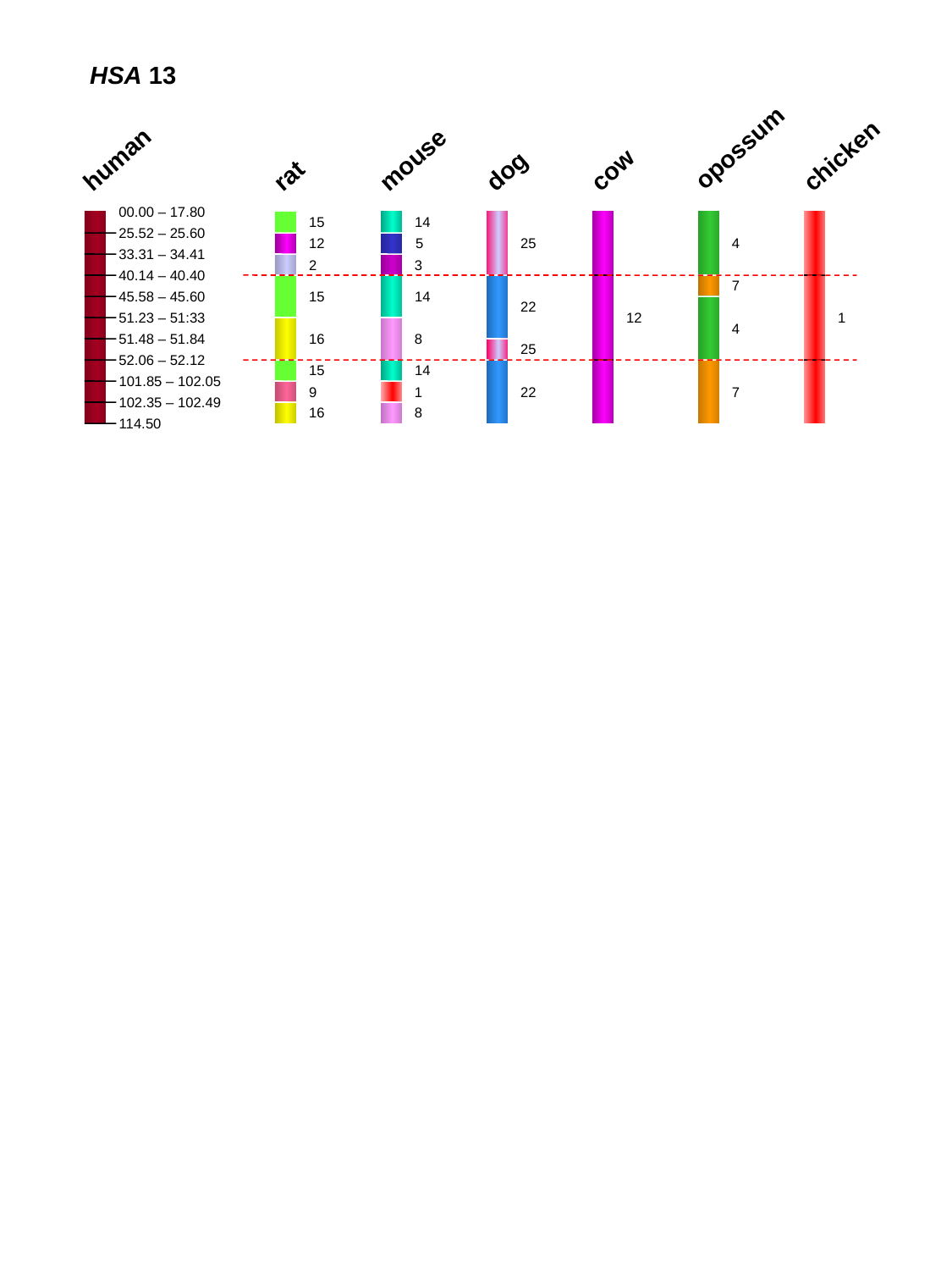

HSA 13
opossum
chicken
human
mouse
cow
dog
rat
00.00 – 17.80
15
14
25.52 – 25.60
12
5
25
4
33.31 – 34.41
2
3
40.14 – 40.40
7
45.58 – 45.60
15
14
22
51.23 – 51:33
12
1
4
51.48 – 51.84
16
8
25
52.06 – 52.12
15
14
101.85 – 102.05
9
1
22
7
102.35 – 102.49
16
8
114.50

## Slide 15
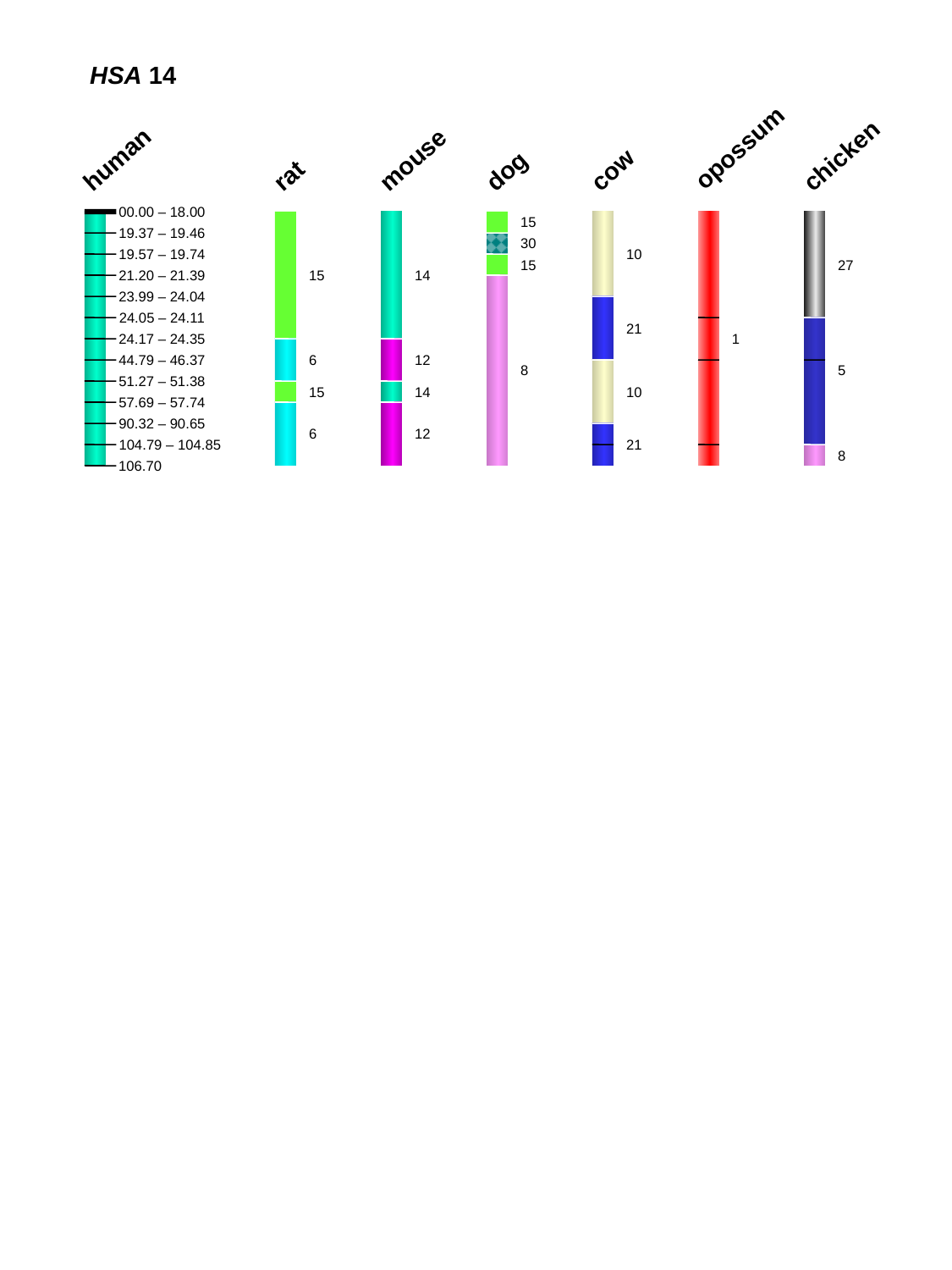

HSA 14
opossum
chicken
human
mouse
cow
dog
rat
00.00 – 18.00
15
19.37 – 19.46
30
19.57 – 19.74
10
15
27
21.20 – 21.39
15
14
23.99 – 24.04
24.05 – 24.11
21
24.17 – 24.35
1
44.79 – 46.37
6
12
8
5
51.27 – 51.38
15
14
10
57.69 – 57.74
90.32 – 90.65
6
12
104.79 – 104.85
21
8
106.70

## Slide 16
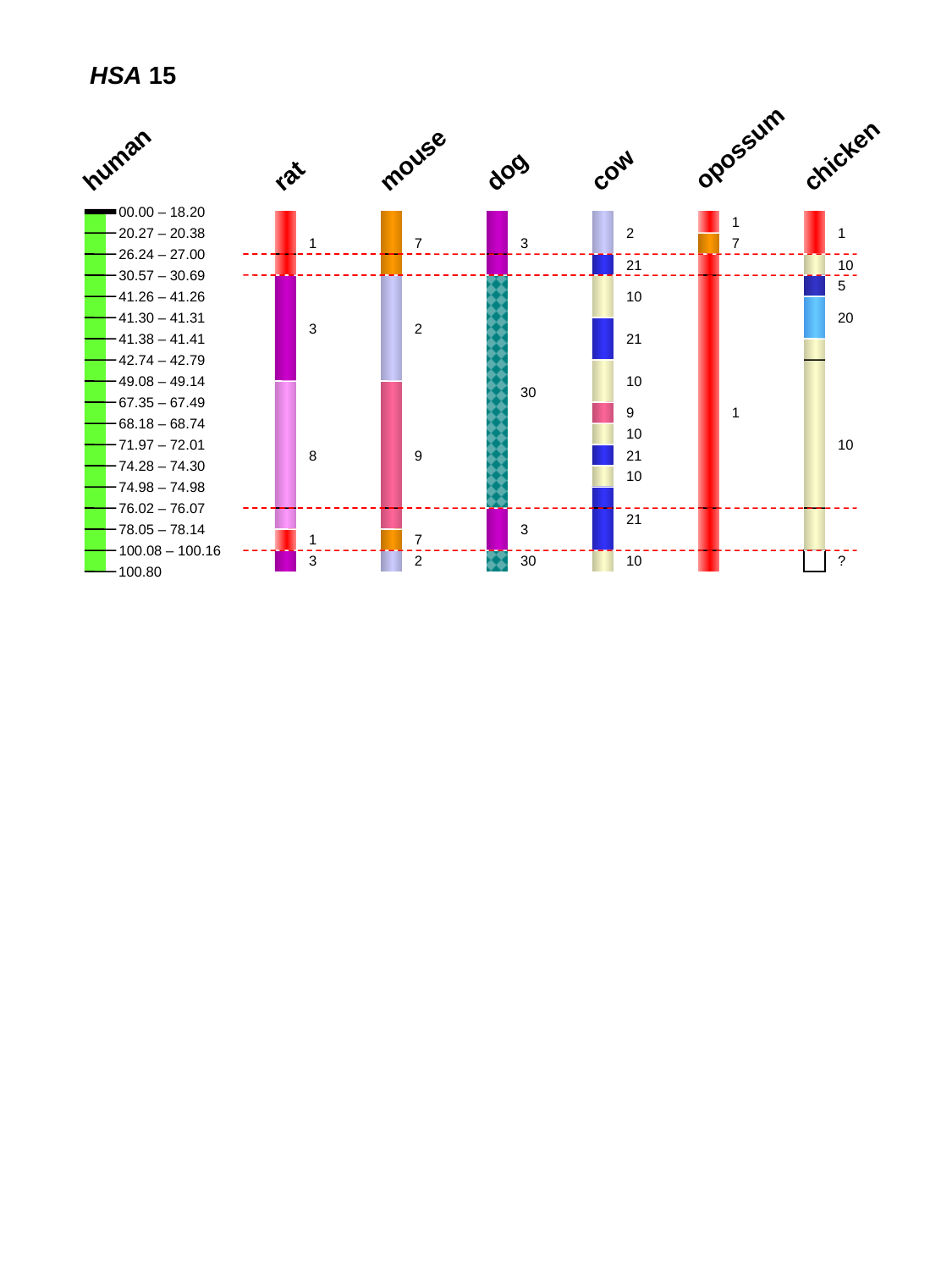

HSA 15
opossum
chicken
human
mouse
cow
dog
rat
00.00 – 18.20
1
20.27 – 20.38
2
1
1
7
3
7
26.24 – 27.00
21
10
30.57 – 30.69
5
41.26 – 41.26
10
41.30 – 41.31
20
3
2
41.38 – 41.41
21
42.74 – 42.79
49.08 – 49.14
10
30
67.35 – 67.49
9
1
68.18 – 68.74
10
71.97 – 72.01
10
8
9
21
74.28 – 74.30
10
74.98 – 74.98
76.02 – 76.07
21
78.05 – 78.14
3
1
7
100.08 – 100.16
3
2
30
10
?
100.80

## Slide 17
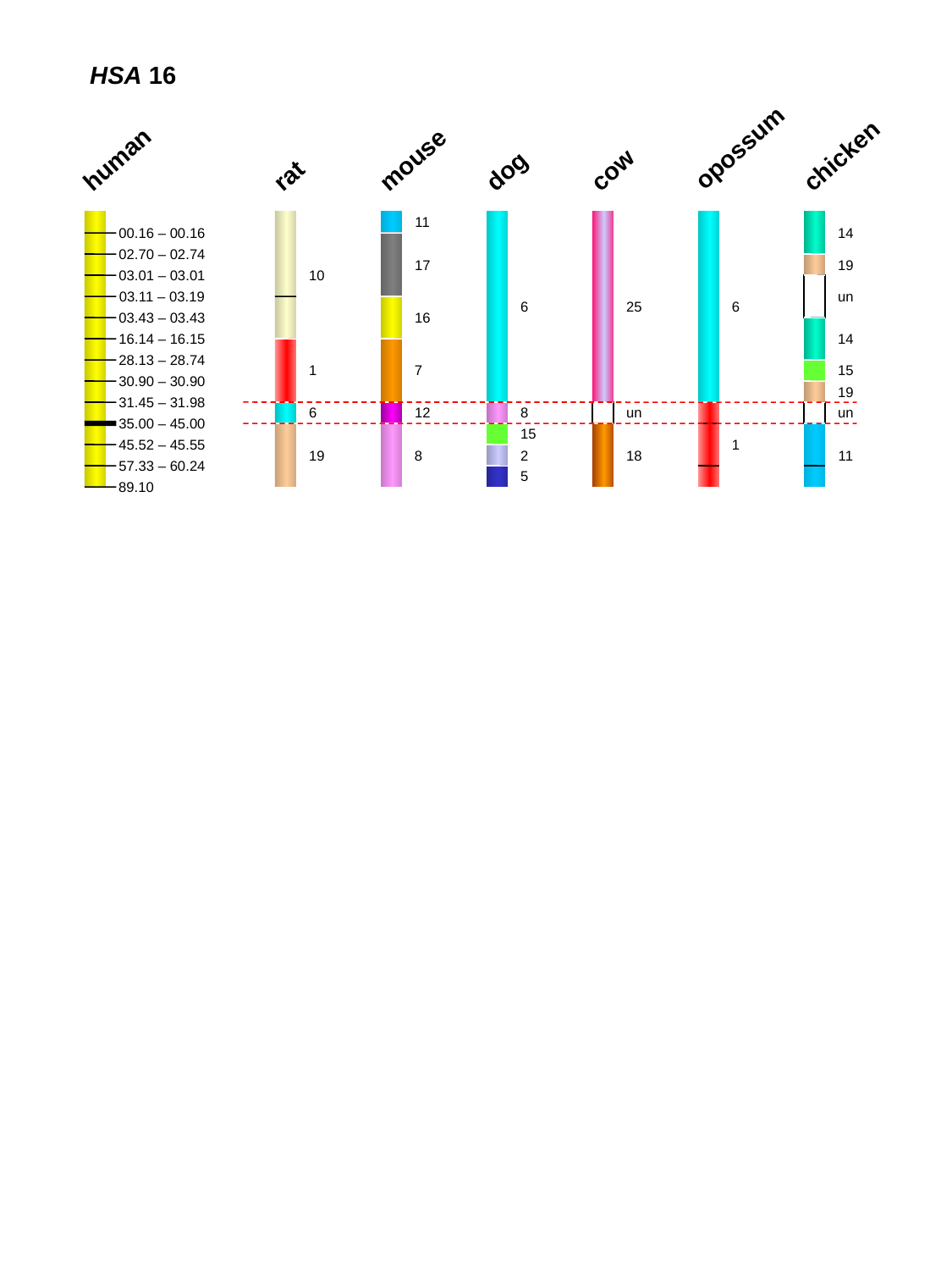

HSA 16
opossum
chicken
human
mouse
cow
dog
rat
11
00.16 – 00.16
14
02.70 – 02.74
17
19
03.01 – 03.01
10
03.11 – 03.19
un
6
25
6
03.43 – 03.43
16
16.14 – 16.15
14
28.13 – 28.74
1
7
15
30.90 – 30.90
19
31.45 – 31.98
6
12
8
un
un
35.00 – 45.00
15
45.52 – 45.55
1
19
8
2
18
11
57.33 – 60.24
5
89.10

## Slide 18
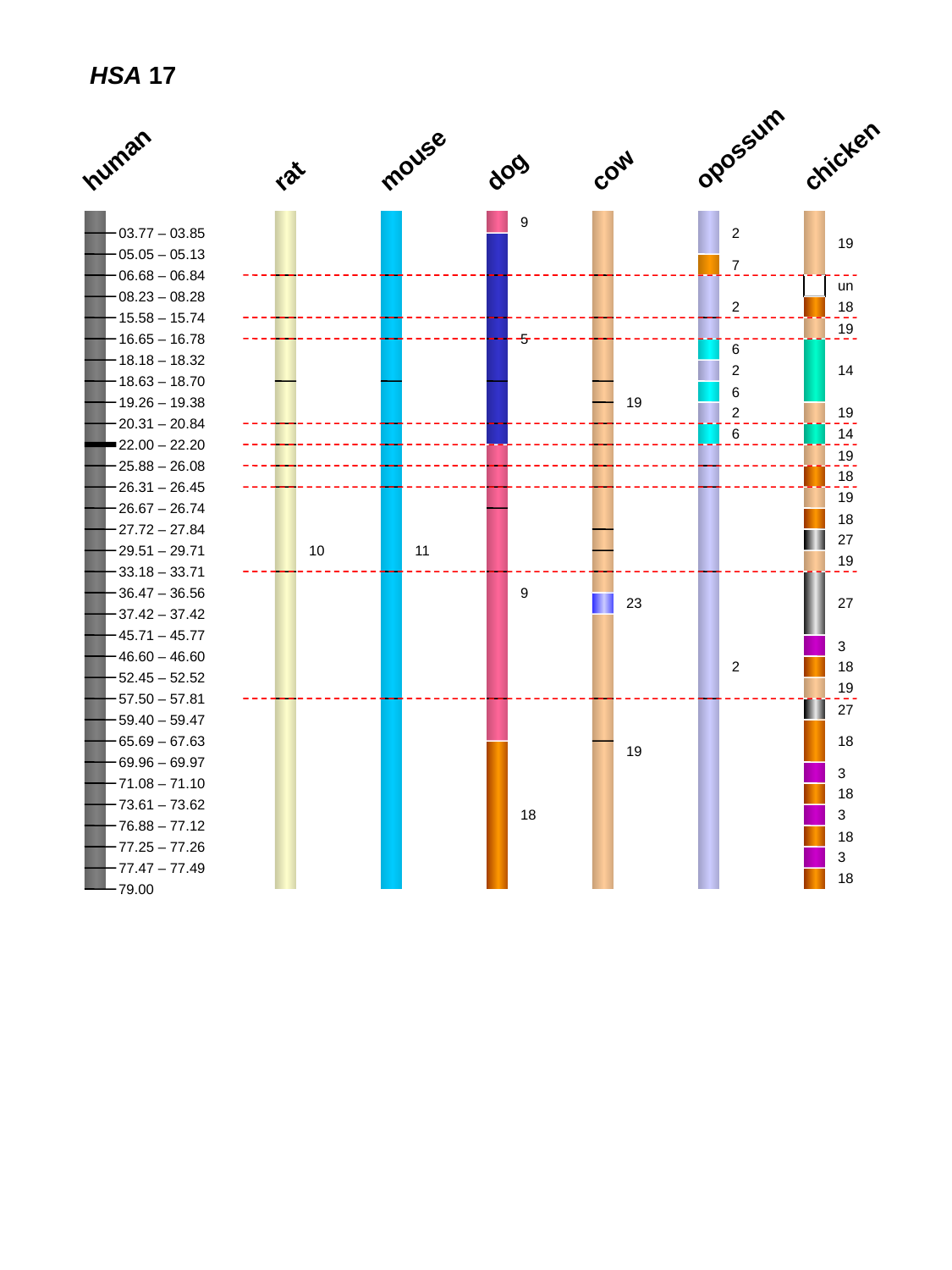

HSA 17
opossum
chicken
human
mouse
cow
dog
rat
9
03.77 – 03.85
2
19
05.05 – 05.13
7
06.68 – 06.84
un
08.23 – 08.28
2
18
15.58 – 15.74
19
16.65 – 16.78
5
6
18.18 – 18.32
2
14
18.63 – 18.70
6
19.26 – 19.38
19
2
19
20.31 – 20.84
6
14
22.00 – 22.20
19
25.88 – 26.08
18
26.31 – 26.45
19
26.67 – 26.74
18
27.72 – 27.84
27
29.51 – 29.71
10
11
19
33.18 – 33.71
36.47 – 36.56
9
23
27
37.42 – 37.42
45.71 – 45.77
3
46.60 – 46.60
2
18
52.45 – 52.52
19
57.50 – 57.81
27
59.40 – 59.47
65.69 – 67.63
18
19
69.96 – 69.97
3
71.08 – 71.10
18
73.61 – 73.62
18
3
76.88 – 77.12
18
77.25 – 77.26
3
77.47 – 77.49
18
79.00

## Slide 19
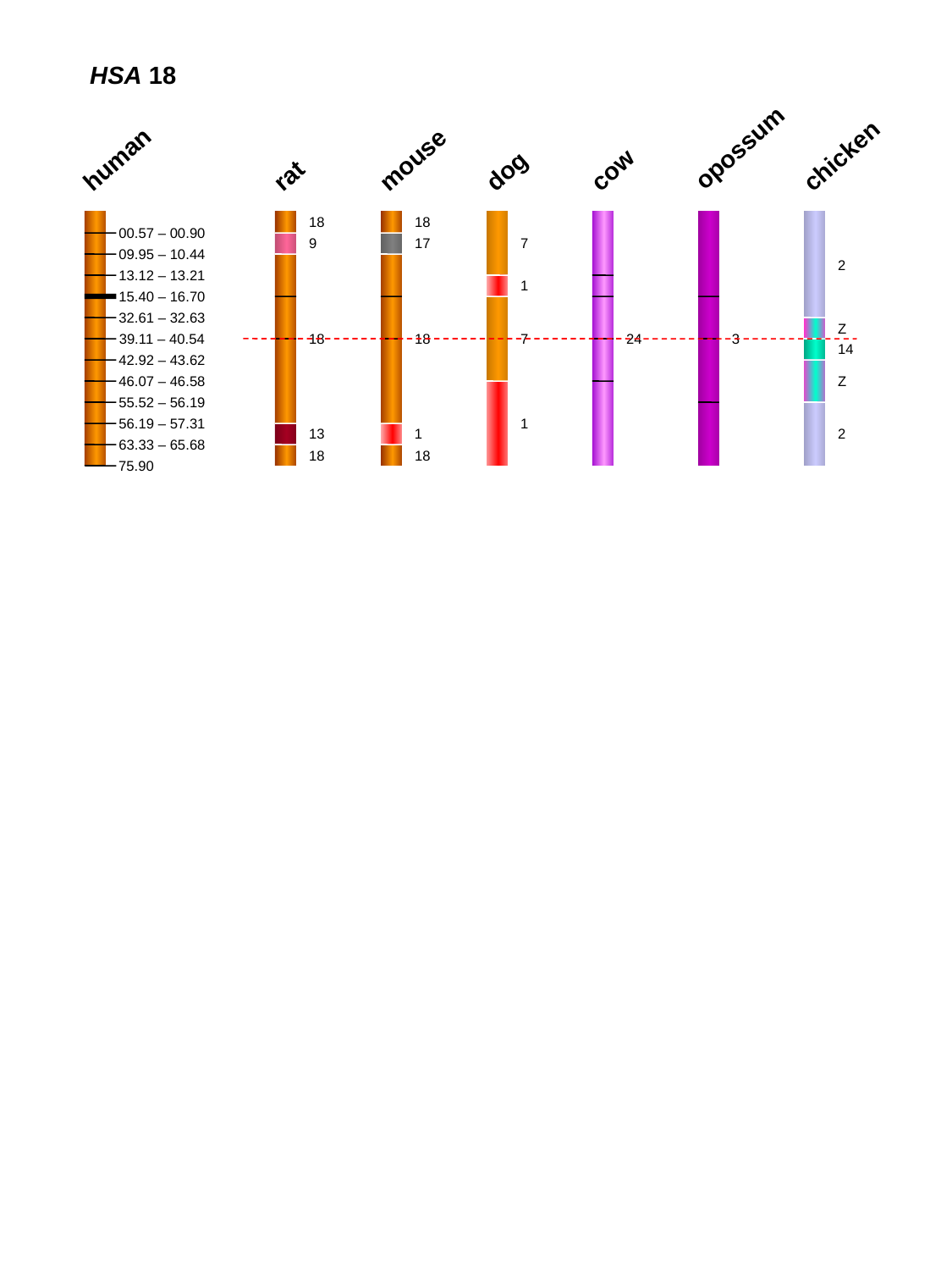

HSA 18
opossum
chicken
human
mouse
cow
dog
rat
18
18
00.57 – 00.90
9
17
7
09.95 – 10.44
2
13.12 – 13.21
1
15.40 – 16.70
32.61 – 32.63
Z
39.11 – 40.54
18
18
7
24
3
14
42.92 – 43.62
46.07 – 46.58
Z
55.52 – 56.19
56.19 – 57.31
1
13
1
2
63.33 – 65.68
18
18
75.90

## Slide 20
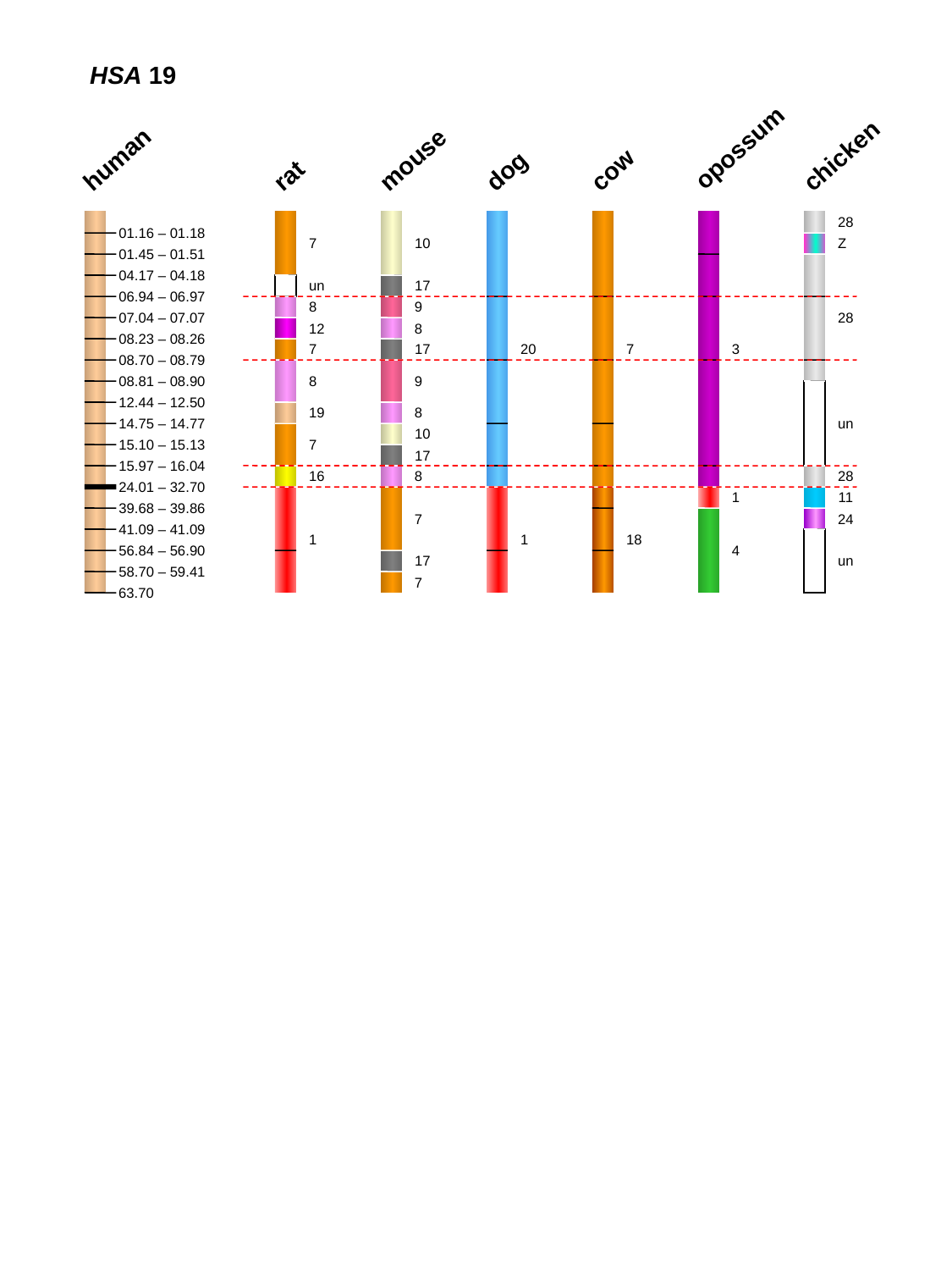

HSA 19
opossum
chicken
human
mouse
cow
dog
rat
28
01.16 – 01.18
7
10
Z
01.45 – 01.51
04.17 – 04.18
un
17
06.94 – 06.97
8
9
07.04 – 07.07
28
12
8
08.23 – 08.26
7
17
20
7
3
08.70 – 08.79
08.81 – 08.90
8
9
12.44 – 12.50
19
8
14.75 – 14.77
un
10
15.10 – 15.13
7
17
15.97 – 16.04
16
8
28
24.01 – 32.70
1
11
39.68 – 39.86
7
24
41.09 – 41.09
1
1
18
56.84 – 56.90
4
17
un
58.70 – 59.41
7
63.70

## Slide 21
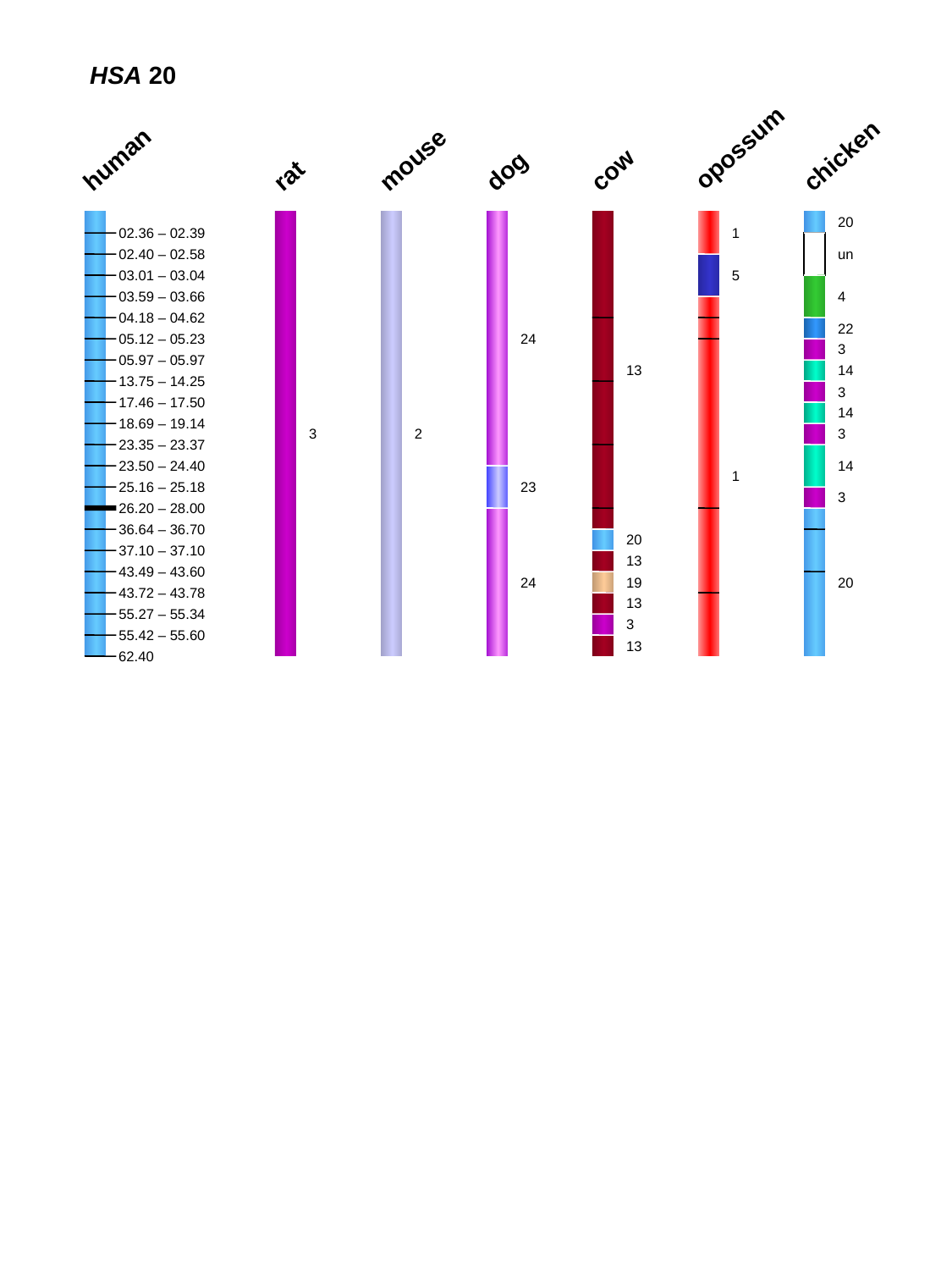

HSA 20
opossum
chicken
human
mouse
cow
dog
rat
20
02.36 – 02.39
1
02.40 – 02.58
un
03.01 – 03.04
5
03.59 – 03.66
4
04.18 – 04.62
22
05.12 – 05.23
24
3
05.97 – 05.97
13
14
13.75 – 14.25
3
17.46 – 17.50
14
18.69 – 19.14
3
2
3
23.35 – 23.37
23.50 – 24.40
14
1
25.16 – 25.18
23
3
26.20 – 28.00
36.64 – 36.70
20
37.10 – 37.10
13
43.49 – 43.60
24
19
20
43.72 – 43.78
13
55.27 – 55.34
3
55.42 – 55.60
13
62.40

## Slide 22
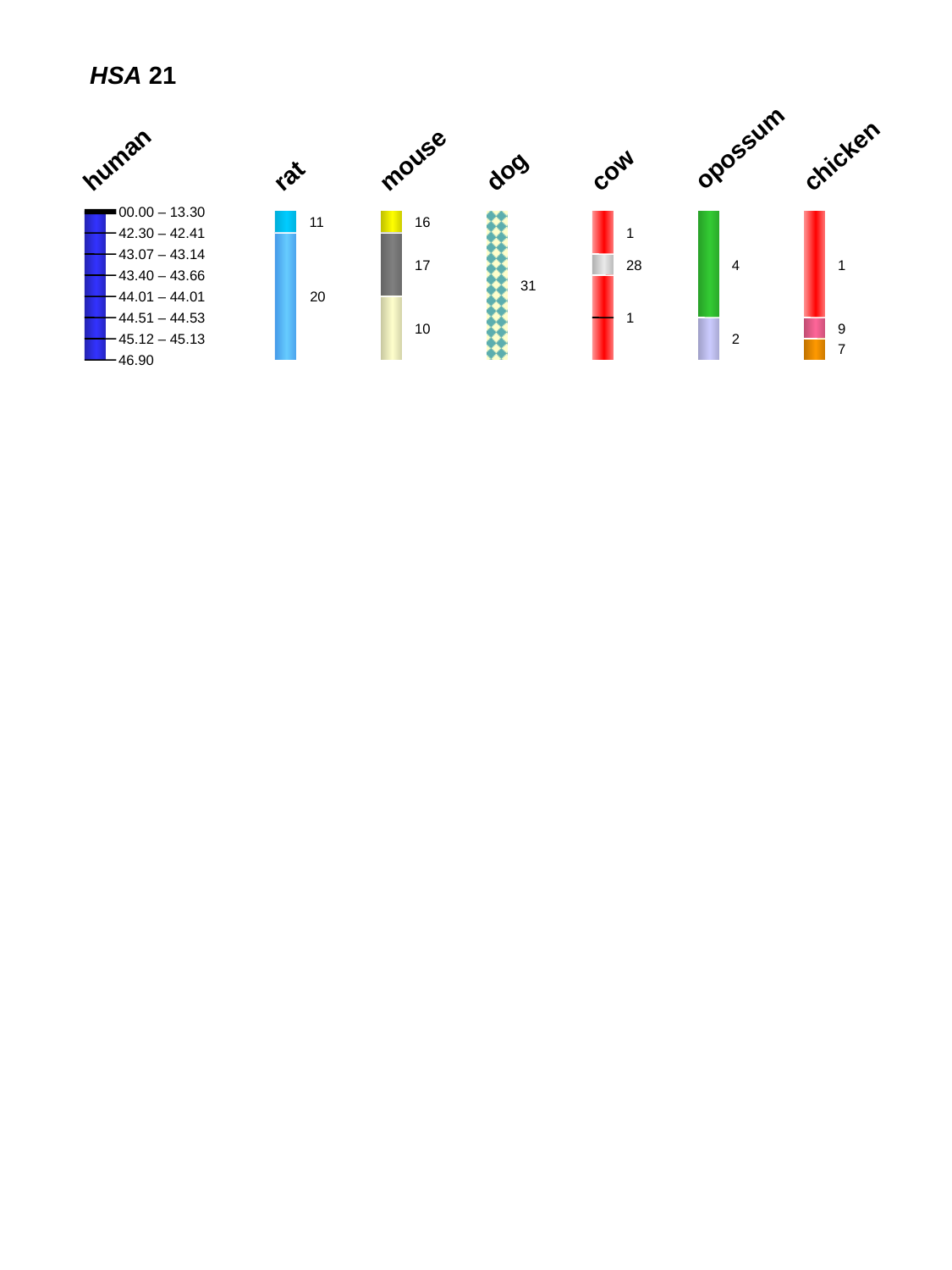

HSA 21
opossum
chicken
human
mouse
cow
dog
rat
00.00 – 13.30
11
16
42.30 – 42.41
1
43.07 – 43.14
17
28
4
1
43.40 – 43.66
31
44.01 – 44.01
20
44.51 – 44.53
1
10
9
45.12 – 45.13
2
7
46.90

## Slide 23
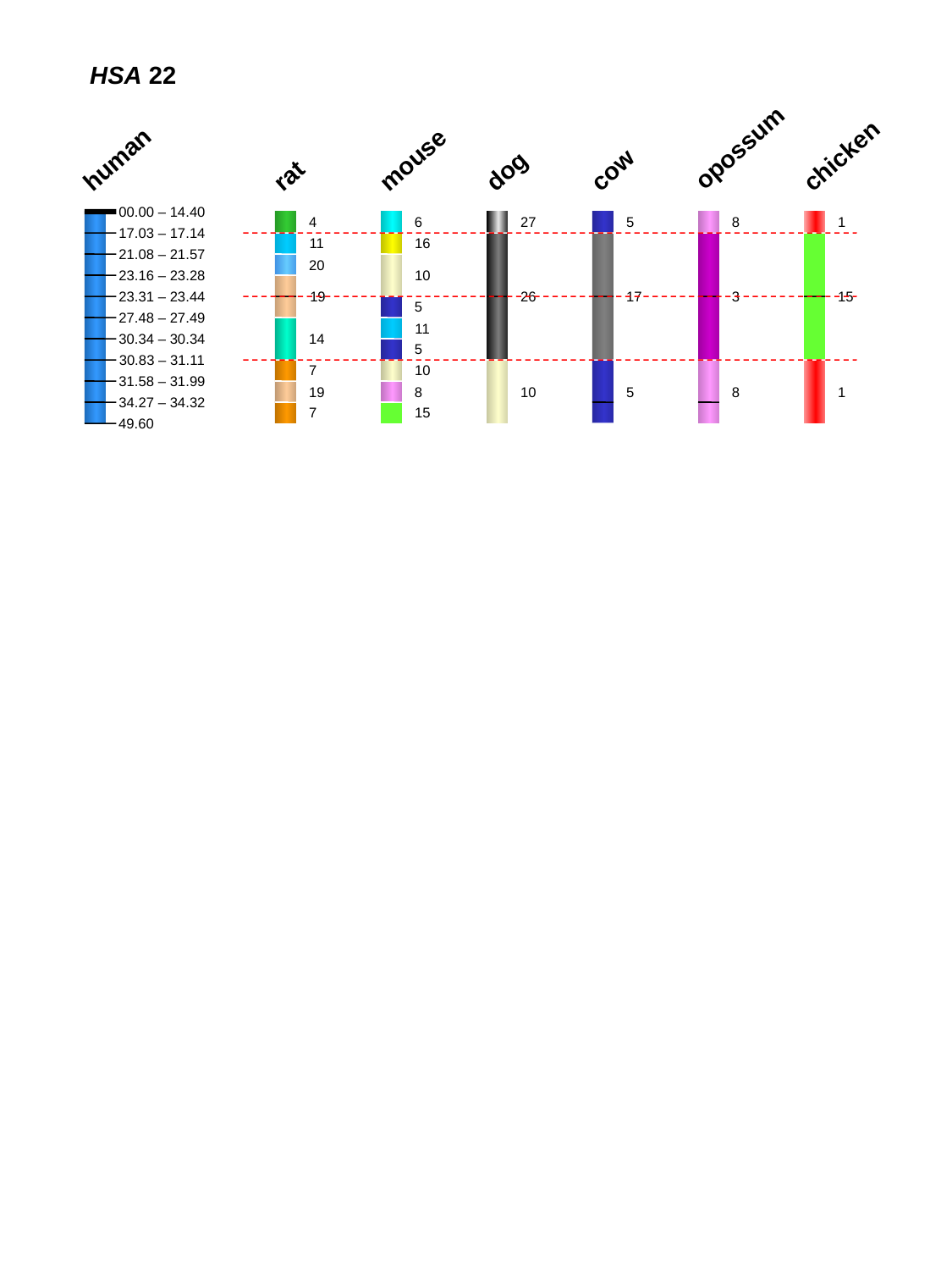

HSA 22
opossum
chicken
human
mouse
cow
dog
rat
00.00 – 14.40
4
6
27
5
8
1
17.03 – 17.14
11
16
21.08 – 21.57
20
23.16 – 23.28
10
23.31 – 23.44
19
26
17
3
15
5
27.48 – 27.49
11
30.34 – 30.34
14
5
30.83 – 31.11
7
10
31.58 – 31.99
19
8
10
5
8
1
34.27 – 34.32
7
15
49.60

## Slide 24
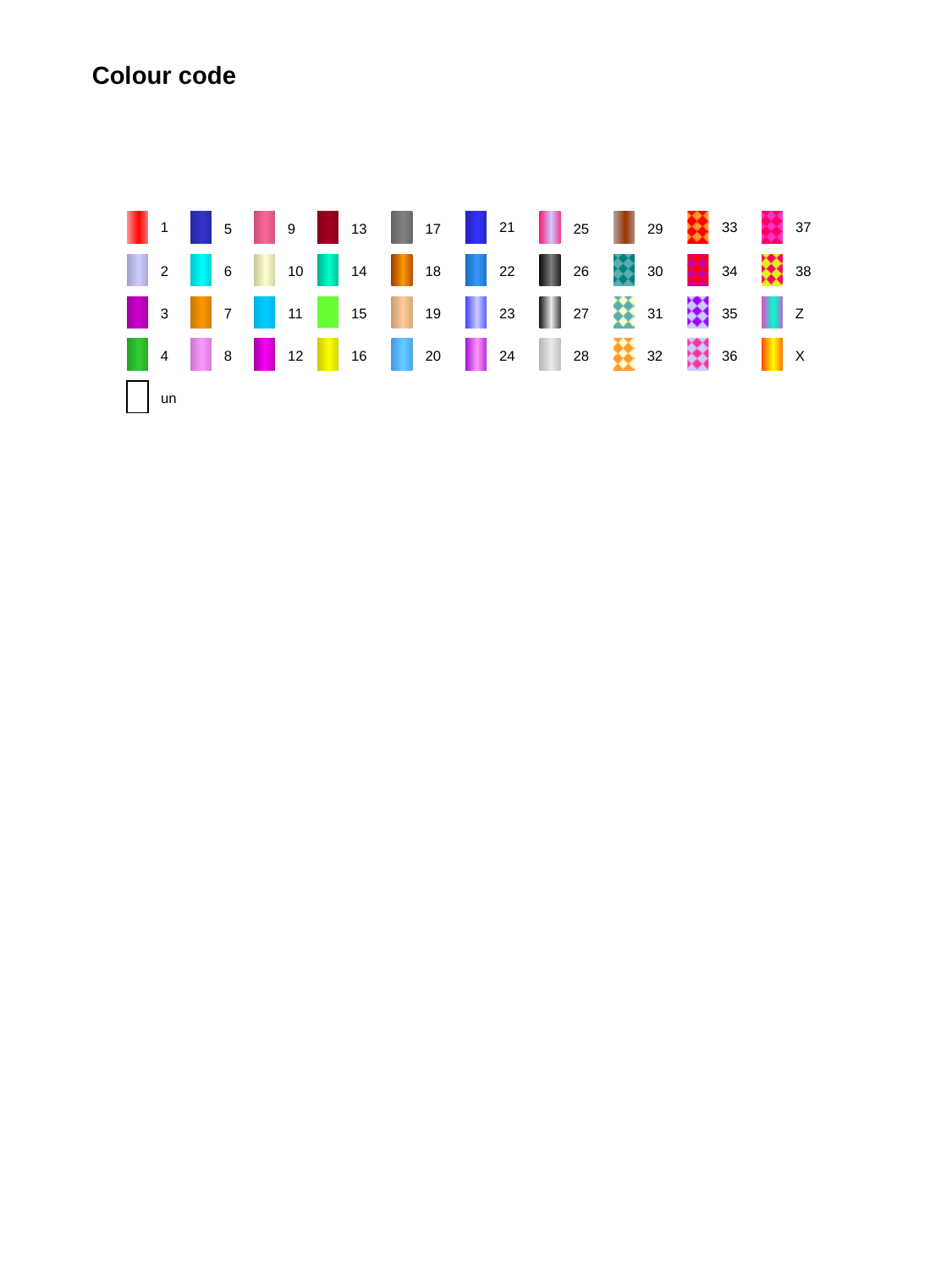

Colour code
1
21
33
37
5
9
13
17
25
29
2
6
10
14
18
22
26
30
34
38
3
7
11
15
19
23
27
31
35
Z
4
8
12
16
20
24
28
32
36
X
un
