# Supplementary material for: Gene synteny comparisons between different vertebrates provide new insights into breakage and fusion events during mammalian karyotype evolution
Source: BMC Evol Biol. 2009 Apr 24;9:84. doi: 10.1186/1471-2148-9-84 (PMC2681463; doi:10.1186/1471-2148-9-84)
Supplement: Additional file 3 — Phylogenetic relationships among the species investigated in this study and the armadillo and the elephant not analysed here (marked with an asterisk) according to Wildman et al. [65]. Scheme to indicate phylogenetic relationships between clades. [file 1471-2148-9-84-S3.ppt]

## Slide 1
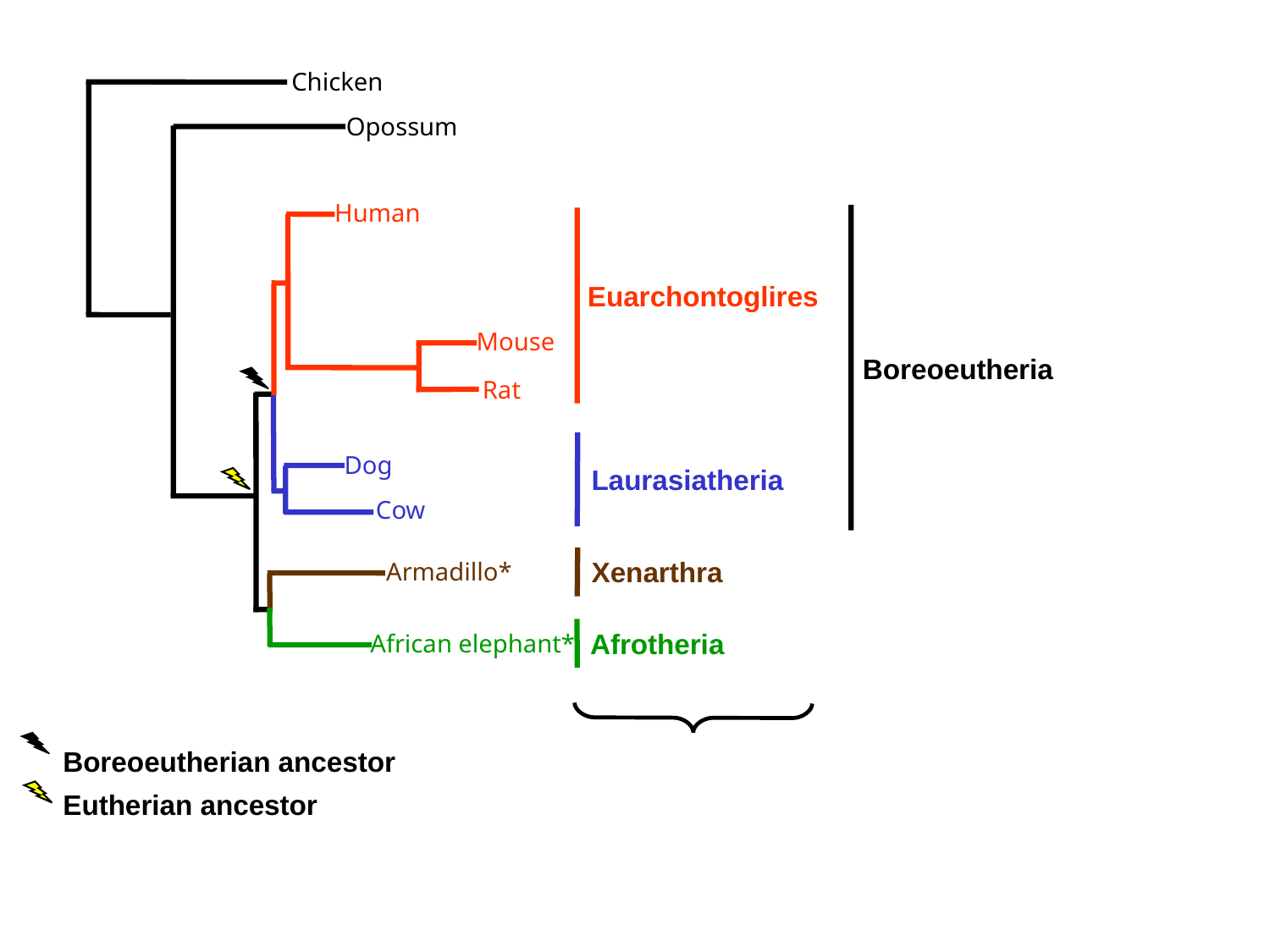

Chicken
Opossum
Human
Euarchontoglires
Mouse
Boreoeutheria
Rat
Dog
Laurasiatheria
Cow
Xenarthra
Armadillo*
Afrotheria
African elephant*
Boreoeutherian ancestor
Eutherian ancestor
Clade
Additional file 3: Phylogenetic relationships among the species investigated in this study and the armadillo and the elephant not analysed here (marked with an asterisk) according to Wildman et al. [65].
