# Supplementary material for: Gene synteny comparisons between different vertebrates provide new insights into breakage and fusion events during mammalian karyotype evolution
Source: BMC Evol Biol. 2009 Apr 24;9:84. doi: 10.1186/1471-2148-9-84 (PMC2681463; doi:10.1186/1471-2148-9-84)
Supplement: Additional file 4 — Average transcript density of all evolutionary breakpoint intervals on human chromosomes. Table indicating the average transcript density of all evolutionary breakpoint intervals. [file 1471-2148-9-84-S4.doc]

**Additional file 4:** Average transcript density of all evolutionary breakpoint intervals on human chromosomes

| Width of analyzed region in Mb | 6 | 4 | 2 | 1.5 | 1 | 0.5 | 0.25 | 0.125 | 0.1 | 0.06 |
| --- | --- | --- | --- | --- | --- | --- | --- | --- | --- | --- |
| Transcripts / Mb^a^ | 14.99 | 15.74 | 16.96 | 17.54 | 18.11 | 18.74 | 19.18 | 20.26 | 20.23 | 19.93 |
| Increase above genome average | 1.27x | 1.34x | 1.44x | 1.49x | 1.54x | 1.59x | 1.63x | 1.72x | 1.72x | 1.70x |

a: The number of transcripts per Mb were determined according to used the Human Transcriptome Map, provided by the UCSC Genome Bioinformatics Project (<http://genome.ucsc.edu/>) according to Versteeg et al. [53].
