# Supplementary material for: Gene synteny comparisons between different vertebrates provide new insights into breakage and fusion events during mammalian karyotype evolution
Source: BMC Evol Biol. 2009 Apr 24;9:84. doi: 10.1186/1471-2148-9-84 (PMC2681463; doi:10.1186/1471-2148-9-84)
Supplement: Additional file 5 — Average transcript density of evolutionary breakpoint intervals mapped to regions under 40 kb (n = 144). Table indicating the average transcript density of evolutionary breakpoints. [file 1471-2148-9-84-S5.doc]

**Additional file 5:** Average transcript density of evolutionary breakpoint intervals mapped to regions under 40 kb (n=144).

| Width of analyzed region (Mb) | 0.125 | 0.1 | 0.06 | 0.04 | 0.02 |
| --- | --- | --- | --- | --- | --- |
| Transcripts/Mb^a^ | 31.04 | 32 | 34 | 34.15 | 29.23 |
| Increase above genome average | 2.64x | 2.72x | 2.89x | 2.90x | 2.49x |

a: The number of transcripts per Mb were determined according to used the Human Transcriptome Map, provided by the UCSC Genome Bioinformatics Project (<http://genome.ucsc.edu/>) according to Versteeg et al. [53].
